# Inspection of Pole-Like Structures Using a Visual-Inertial Aided VTOL Platform with Shared Autonomy

**DOI:** 10.3390/s150922003

**Published:** 2015-09-02

**Authors:** Inkyu Sa, Stefan Hrabar, Peter Corke

**Affiliations:** 1 Science and Engineering Faculty, Queensland University of Technology, Brisbane 4000, Australia; E-Mail: peter.corke@qut.edu.au; 2 CSIRO Digital Productivity, Brisbane 4069, Australia; E-Mail: Stefan.Hrabar@csiro.au

**Keywords:** aerial robotics, pole inspection, visual servoing, shared autonomy

## Abstract

This paper presents an algorithm and a system for vertical infrastructure inspection using a vertical take-off and landing (VTOL) unmanned aerial vehicle and shared autonomy. Inspecting vertical structures such as light and power distribution poles is a difficult task that is time-consuming, dangerous and expensive. Recently, micro VTOL platforms (*i.e.*, quad-, hexa- and octa-rotors) have been rapidly gaining interest in research, military and even public domains. The unmanned, low-cost and VTOL properties of these platforms make them ideal for situations where inspection would otherwise be time-consuming and/or hazardous to humans. There are, however, challenges involved with developing such an inspection system, for example flying in close proximity to a target while maintaining a fixed stand-off distance from it, being immune to wind gusts and exchanging useful information with the remote user. To overcome these challenges, we require accurate and high-update rate state estimation and high performance controllers to be implemented onboard the vehicle. Ease of control and a live video feed are required for the human operator. We demonstrate a VTOL platform that can operate at close-quarters, whilst maintaining a safe stand-off distance and rejecting environmental disturbances. Two approaches are presented: Position-Based Visual Servoing (PBVS) using an Extended Kalman Filter (EKF) and estimator-free Image-Based Visual Servoing (IBVS). Both use monocular visual, inertia, and sonar data, allowing the approaches to be applied for indoor or GPS-impaired environments. We extensively compare the performances of PBVS and IBVS in terms of accuracy, robustness and computational costs. Results from simulations and indoor/outdoor (day and night) flight experiments demonstrate the system is able to successfully inspect and circumnavigate a vertical pole.

## 1. Introduction

This paper presents an inspection system based on a vertical take-off landing (VTOL) platform and shared autonomy. The term “shared autonomy” indicates that the major fraction of control is accomplished by a computer. The operator’s interventions for low-level control are prohibited but the operator provides supervisory high-level control commands such as setting the goal position. In order to perform an inspection task, a VTOL platform should fly in close proximity to the target object being inspected. This close-quarters flying does not require global navigation (explorations of large known or unknown environments) but instead requires local navigation relative to the specific geometry of the target, for instance, the pole of a streetlight. Such a system allows an unskilled operator to easily and safely control a VTOL platform to examine locations that are otherwise difficult to reach. For example, it could be used for practical tasks such as inspecting for bridge or streetlight defects. Inspection is an important task for the safety of structures but is a dangerous and labor intensive job. According to the US Bureau of Transportation Statistics, there are approximately 600,000 bridges in the United States and 26% of them require inspections. Echelon, an electricity company, reported that there are 174.1 million streetlights in the US, Europe, and UK [1]. These streetlights also require inspections every year. These tasks are not only high risk for the workers involved but are slow, labour intensive and therefore expensive. VTOL platforms can efficiently perform these missions since they can reach places that are high and inaccessible such as the outsides of buildings (roof or wall), high ceilings, the tops of poles and so on. However, it is very challenging to use these platforms for inspection because there is insufficient room for error and high-level pilot skills are required as well as line-of-sight from pilot to vehicle. This paper is concerned with enabling low-cost semi-autonomous flying robots, in collaboration with low-skilled human operators, to perform useful tasks close to objects.

Multi-rotor VTOL micro aerial vehicles (MAVs) have been popular research platforms for a number of years due to advances in sensor, battery and integrated circuit technologies. The variety of commercially-available platforms today is testament to the fact that they are leaving the research labs and being used for real-world aerial work. These platforms are very capable in terms of their autonomous or attitude stabilized flight modes and the useful payloads they can carry. Arguably the most common use is for the collection of aerial imagery, for applications such as mapping, surveys, conservation and infrastructure inspection. Applications such as infrastructure inspection require flying at close-quarters to vertical structures in order to obtain the required images. Current regulations require the MAV’s operator to maintain visual line-of-sight contact with the aircraft, but even so it is an extremely challenging task for the operator to maintain a safe, fixed distance from the infrastructure being inspected. From the vantage point on the ground it is hard to judge the stand-off distance, and impossible to do so once the aircraft is obscured by the structure. The problem is exacerbated in windy conditions as the structures cause turbulence. The use of First-Person View (FPV) video streamed live from the platform can help with situational awareness, but flying close to structures still requires great skill and experience by the operator and requires a reliable low-latency high-bandwidth communication channel. It has been found that flight operations near vertical structures is best performed by a team of three people: a skilled pilot, a mission specialist, and a flight director [2]. For small VTOL MAVs to truly become ubiquitous aerial imaging tools that can be used by domain experts rather than skilled pilots, their level of autonomy must be increased. One avenue to increased autonomy of a platform is through shared autonomy, where the majority of control is accomplished by the platform, but operator input is still required. Typically, the operator is relieved from the low-level relative-control task which is better performed by a computer, but still provides supervisory high-level control commands such as a goal position. We employ this shared autonomy approach for the problem of MAV-based vertical infrastructure inspections.

**Figure 1:**
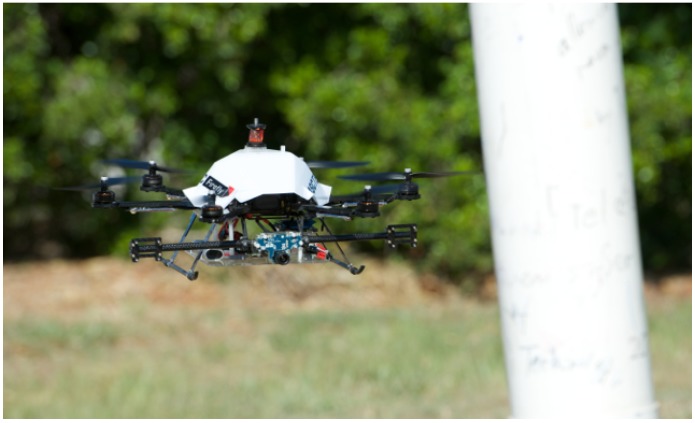
The vertical take-off and landing (VTOL) platform used for our pole inspection experiments. It includes a front-facing camera, downward-facing ultrasonic sensor and an onboard inertial measurement unit (IMU) for attitude control. All processing occurs onboard using a quad-core Acorn Risc Machine (ARM) Cortex-A9 processor.

It is useful for an operator to be able to “guide” the MAV in order to obtain the required inspection viewpoints without the cognitive workload of “piloting” it. We provide the additional autonomy needed by implementing visual plus inertial-based pole-relative hovering as well as object circumnavigation shown in Figure 1. By tracking the two edges of the pole in the image and employing Position-Based Visual Servoing (PBVS) or Image-Based Visual Servoing (IBVS), the platform is able to maintain a user specified distance from the pole and keep the camera oriented towards the pole. The operator is also able to control the height and yaw of the platform. Since the pole is kept centred in the image, a yaw rate control command results in an orbit about the pole. A cylindrical workspace around the pole is therefore available to the operator for manoeuvres.

### 1.1. Related Work

#### 1.1.1. Climbing Robots for Inspection Tasks

As mentioned before, inspecting structures, such as light and power distribution poles is a time-consuming, dangerous and expensive task with high operator workload. The options for inspecting locations above the ground are rather limited, and all are currently cumbersome. Ladders can be used up to a height of 10–15 m but are quite dangerous: each year 160 people are killed and 170,000 injured in falls from ladders in the United States [3]. Cherry pickers require large vehicle access, sufficient space to operate and considerable setup time.

Robotics and mechatronics researchers have demonstrated a variety of climbing robots. Considerable growth in sensor and integrated circuit technology has accelerated small and lightweight robotics development. Typically, these robots are inspired by reptiles, mammals and insects, and their type of movement varies between sliding, swinging, extension and jumping.

The flexible mechatronic assistive technology system (MATS) robot has five degrees of freedom (DOF) and a symmetrical mechanism [4]. The robot shows good mobility features for travel, however, it requires docking stations that are attached to the wall, ceiling, or anywhere the robot is required to traverse. The bio-mimicking gekko robot, StickyBot [5], does not require docking stations since it has hierarchical adhesive structures under its toes to hold itself on any kind of surface. It has, however, limitations for payload and practical applications. A bridge cable inspection robot [6] is more applicable than the StickyBot in terms of its climbing speed and payload carrying ability. It climbs the cables by means of wheels which remain in contact with the cable for traction. A climbing robot with legged locomotion was developed by Haynes *et al.* [7]. This robot was designed for high-speed climbing of a uniformly convex cylindrical structure, such as a telephone or electricity pole. NASA’s Jet Propulsion Laboratory recently demonstrated a rock climbing robot utilizing a hierarchical array of claws (called microspines) to create an attachment force of up to 180 N normal to the surface [8]. This robot also can drill a hole with a self-contained rotary percussive drill while it is attached to the surface.

Since climbing robots are in contact with the surface they can perform contact-based high-precision inspection with high performance sensors. They are also able to perform physical actions on the surface, not just inspections [9]. These climbing robots could not only replace a worker undertaking risky tasks in a hazardous environment but also increase the efficiency of such tasks. Climbing robots, however, require complex mechanical designs and complicated dynamic analysis. Their applications are also limited to structures with specific shapes and surface materials. They require setup time and climb slowly, so the inspection task can be time-consuming.

#### 1.1.2. Flying Robots for Inspection Tasks

VTOL platforms on the other hand offer a number of advantages when used for infrastructure inspection. They have relatively simple mechanical designs (usually symmetric) which require a simple dynamic analysis and controller. VTOL platforms can ascend quickly to the required height and can obtain images from many angles regardless of the shape of the structure. Recent advanced sensor, integrated circuit and motor technologies allow VTOL platforms to fly for a useful amount of time while carrying inspection payloads. Minimal space is required for operations and their costs are relatively low. The popularity of these platforms means that hardware and software resources are readily available [10].

These advantages have accelerated the development of small and light-weight flying robotics for inspection. Voigt *et al.* [11] demonstrated an embedded stereo-camera based egomotion estimation technique for the inspection of structures such as boilers and general indoor scenarios. The stereo vision system provides a relative pose estimate between the previous and the current frame and this is fed into an indirect Extended Kalman Filter (EKF) framework as a measurement update. The inertial measurements such as linear accelerations and rotation rates played important roles in the filter framework. States, (position, orientation, bias, and relative pose) were propagated with IMU measurements through a prediction step and the covariance of the predicted pose were exploited to determine a confidence region for feature searching in the image plane. This allowed feature tracking on scenes with repeating textures (perception aliasing), increased the total number of correct matches (inliers), and efficiently rejected outliers with reasonable computation power. They evaluated the proposed method on several trajectories with varying flight velocities. The results presented show the vehicle is capable of impressively accurate path tracking. However, flights tests were performed indoors in a boiler mock-up environment where disturbances are not abundant, and using hand-held sequences from an office building dataset. Based on this work, Burri *et al.* [12] and Nikolic *et al.* [13] show visual inspection of a thermal power plant boiler system using a quadrotor. They developed a Field Programmable Gate Array (FPGA) based visual-inertial stereo Simultaneous Localization and Mapping (SLAM) sensor with state updates at 10 Hz. A model predictive controller (MPC) is used for closed loop control in industrial boiler environments. In contrast to their work, we aim for flights in outdoor environments where disturbances such as wind gusts are abundant and the scenes include natural objects.

Ortiz *et al.* [14] and Eich *et al.* [15] introduced autonomous vessel inspection using a quadrotor platform. A laser scanner is utilized for horizontal pose estimation with Rao-Blackwellized particle filter based SLAM (GMapping), and small mirrors reflected a few of the horizontal beams vertically downwards for altitude measurement. These technologies have been adopted from the 2D ground vehicle SLAM solution into aerial vehicle research [16] and often incorporated within a filter framework for fast update rates and accurate state estimation [17]. While such methods are well-established and optimized open-source software packages are available, one of the main drawbacks is the laser scanner. Compared to monocular vision, a laser scanner is relatively heavy and consumes more power, which significantly decreases the total flight time. Instead, we propose a method using only a single light-weight camera, a geometric model of the target object, and a single board computer for vertical structure inspection tasks.

### 1.2. Contributions and Overview

This paper contributes to the state-of-the-art in aerial inspections by addressing the limitations of existing approaches presented in Section 1.1 with the proposed high performance vertical structure inspection system. In this paper, we make use of our previous developed robust line feature tracker [18] as a front-end vision system, and it is summarized in Section 2.2. A significant difference to our previous works [19,20,21] in which different flying platforms had been utilized is the integration of both PBVS and IBVS systems on the same platform. By doing so, we are able to compare both systems quantitatively. We also conduct experiments where a trained pilot performs the same tasks using manual flight and with the aid of PBVS and IBVS and demonstrate the difficulty of the tasks. For evaluation, motion capture systems, a laser tracker, and hand-annotated images are used. Therefore, the contributions of this paper are:

- The development of onboard flight controllers using monocular visual features (lines) and inertial sensing for visual servoing (PBVS and IBVS) to enable VTOL MAV close quarters manoeuvring.
- The use of shared autonomy to permit an un-skilled operator to easily and safely perform MAV-based pole inspections in outdoor environments, with wind, and at night.
- Significant experimental evaluation of state estimation and control performance for indoor and outdoor (day and night) flight tests, using a motion capture device and a laser tracker for ground truth. Video demonstration [22].
- A performance evaluation of the proposed systems in comparison to skilled pilots for a pole inspection task.

The remainder of the paper is structured as follows: Section 2 describes the coordinate system definition used in this paper, and the vision processing algorithms for fast line tracking. Section 3 and Section 4 present the PBVS and IBVS control structures which are developed for the pole inspection scenario, and with validation through simulation. Section 5 presents the use of shared autonomy and we present our extensive experimental results in Section 6. Conclusions are drawn in Section 7.

## 2. Coordinate Systems and Image Processing

### 2.1. Coordinate Systems

**Figure 2:**
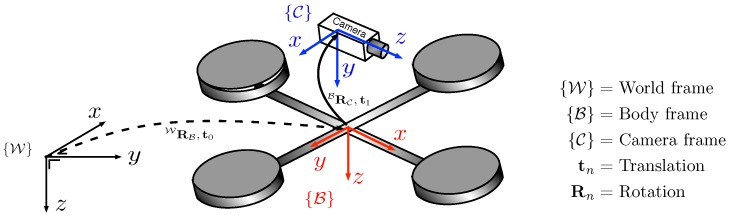
Coordinate systems: body {B}, world {W}, and camera {C}. Transformation between {B} and {C} is constant whereas {B} varies as the quadrotor moves. ${}^{C}R_{B}$ rotates a vector defined with respect to {B} to a vector with respect to {C}.

We define three right-handed frames: world {W}, body {B} and camera {C} which are shown in Figure 2. Note that both {W} and {B} have their z-axis downward while {C} has its z-axis (camera optical axis) in the horizontal plane of the propellers and pointing in the vehicle’s forward direction. We define the notation ${}^{a}R_{b}$ which rotates a vector defined with respect to frame {b} to a vector with respect to {a}.

### 2.2. Image Processing for Fast Line Tracking

Our line tracker is based on tracking the two edges of the pole over time. This is an appropriate feature since the pole will dominate the scene in our selected application. There are many reported line extraction algorithms such as Hough transform [23] and other linear feature extractors [24] but these methods are unsuitable due to their computational complexity. Instead we use a simple and efficient line tracker inspired by [25]. The key advantage of this algorithm is its low computation requirement. For 320×240 pixel images every iteration is finished in <16 ms and uses only 55% of the CPU quad-core ARM Cortex-A9.

#### 2.2.1. 2D and 3D Line Models

A 3D line can be described using various parameterizations including two 3D points, the intersection of two 3D planes, closest point with direction or two projections. These representations vary in terms of their properties including completeness, reprojection characteristics with a perspective camera and the number of internal constraints [26]. *Plücker coordinates* [27] have been widely used in the computer vision and the robotic community for 3D line reconstruction [28], line based visual servoing [29] and SLAM [30]. Plücker coordinates describe a line joining the two 3D points ${}^{W}A$ and ${}^{W}B$ $\in R^{3}$ in the world frame according to

(1) $$\begin{array}{c} {}^{W}L={}^{W}\tilde{A}{}^{W}{\tilde{B}}^{T}-{}^{W}\tilde{B}{}^{W}{\tilde{A}}^{T} \end{array}$$

where ${}^{W}L$ is a Plücker matrix $\in R^{4\times 4}$. The tilde denotes the homogeneous form of the point ($\in P^{3}$).

Consider a perspective projection represented by a camera matrix (intrinsic and extrinsic) $C\left(\xi_{C}\right)\in R^{3\times 4}$

(2) $$\begin{array}{cc} C(\xi_{C}) & =\left[\begin{array}{c} f_{x}\;\;0\;\;u_{0}\\ 0\;\;f_{y}\;\;v_{0}\\ 0\;\;0\;\;\;\;1 \end{array}\right]\left[\begin{array}{c} 1\;0\;0\;0\\ 0\;1\;0\;0\\ 0\;0\;1\;0 \end{array}\right]\xi_{C}^{-1}\\ & =KP_{0}\xi_{C}^{-1} \end{array}$$

where $\xi_{C}\in SE\left(3\right)$ is the camera pose with respect to the world coordinate frame, $f_{x}$ and $f_{y}$ are focal lengths, $u_{0}$ and $v_{0}$ are the coordinates of the principal point.

**Figure 3:**
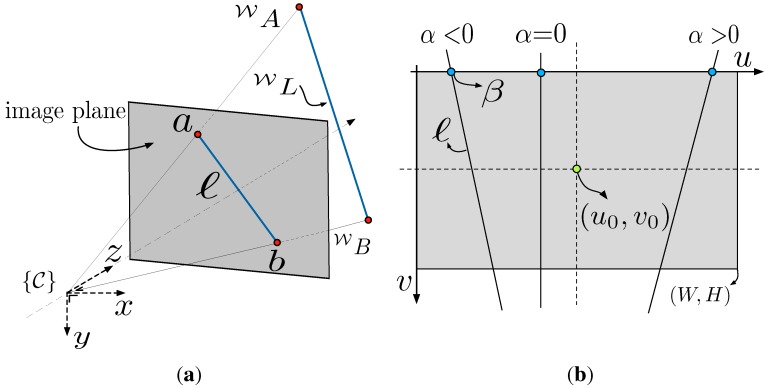
(**a**) Perspective image of a line ${}^{W}L$ in 3D space. *a* and *b* are projections of the world point and ***ℓ*** is a line on the image plane; (**b**) Image plane representation of slope (*α*) and intercept (*β*).

The 3D line ${}^{W}L$ is projected to a 2D line on the camera image plane by

(3) $$\begin{array}{cc} {\left[\ell \right]}_{\times }= & C\left(\xi_{C}\right)\;{}^{W}L\;C{\left(\xi_{C}\right)}^{T} \end{array}$$

where ${\left[\ell \right]}_{\times }$ is a skew-symmetric matrix and $\ell =(\ell_{1},\ell_{2},\ell_{3})$ is the homogeneous line equation on the image plane

(4) $$\begin{array}{c} \ell_{1}u+\ell_{2}v+\ell_{3}=0 \end{array}$$

where *u* and *v* are the horizontal and vertical image plane coordinates respectively (see Figure 3). We reparameterize the line as

(5) $$\begin{array}{c} \ell ={\left[\alpha ,\beta \right]}^{T},\;\mathrm{where}\;\;\alpha =\frac{\ell_{1}}{\ell_{2}},\;\beta =\frac{-\ell_{3}}{\ell_{2}} \end{array}$$

and *α* is the slope and *β* is the x-axis intercept (in pixels), see Figure 3b. Note that this parameterization is the $\frac{\pi }{2}$ rotated form of the conventional 2D line equation, in order to avoid the singular case for a vertical line. There is a singularity for a horizontal line ($\ell_{2}=0$) but we do not expect this in our application.

#### 2.2.2. Line Prediction and Tracking

We use a linear feature velocity model for line prediction

(6) $$\begin{array}{c} {\hat{\ell }}_{k+1}=\ell_{k}+\Delta {\dot{\ell }}_{k} \end{array}$$

where *k* is the timestep, ${\hat{\ell }}_{k+1}$ is the predicted line in the image plane, ${\dot{\ell }}_{k}$ is the feature velocity, $\ell_{k}$ is the previously observed feature and Δ is the sample time. In order to calculate feature velocity, we compute an *image Jacobian*, $J_{l}$, which describes how a line moves on the image plane as a function of camera spatial velocity $\nu ={\left[{}^{C}\dot{x},{}^{C}\dot{y},{}^{C}\dot{z},{}^{C}\omega_{x},{}^{C}\omega_{y},{}^{C}\omega_{z}\right]}^{T}$[31].

(7) $${\dot{\ell }}_{k}=J_{lk}\nu_{k}$$

This image Jacobian is the derivative of the 3D line projection function with respect to camera pose, and for the line parameterization of Equation (5) $J_{l}\in R^{2\times 6}$.

**Figure 4:**
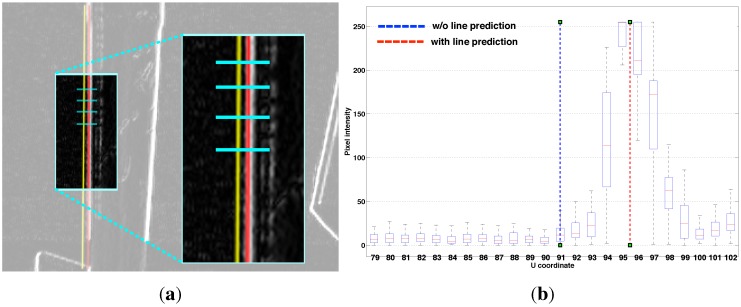
(**a**) The image at time *k* is shown with the tracked line from time k-1 (without prediction case in yellow) and the predicted line from time k-1 (with prediction case in red). We search for maxima along 60 horizontal search lines (cyan), and each is 24 pixels wide; (**b**) The predicted line is close to the maxima whereas there is 4.5 pixel offset without prediction.

The line tracker has two phases: bootstrapping and tracking. The computationally expensive Canny edge detection and Hough transform are utilized only once for bootstrapping. The tracking phase is invoked while the vehicle is flying. There are two steps in the tracking phase: line searching and line model fitting. Horizontal gradient (Sobel kernel) images are computed which emphasise vertical lines in the scene. We sample at 60 points uniformly distributed vertically along the predicted lines. We then compute maxima along a fixed-length horizontal scan line centred on each of these points, see Figure 4a. The horizontal scan line length is empirically set to 24 pixels. These maxima are input to a line fitting algorithm using RANSAC [32], to update the line model for the next iteration.

For vision-based control methods it is critical to have feature tracking that is robust to agile camera motion and lighting condition changes. To handle agile motion we make use of inertial measurements, acceleration and angular velocity in the body coordinate frame, to predict where the feature will be in the image plane for the next frame. Figure 4 shows an example of the prediction result. At this moment, the camera had an acceleration of 1.7 m/s^2^ and rotation rate of 19 °/s. The yellow and red lines in Figure 4a denote the cases without and with prediction respectively, and shows qualitatively that the red line is closer to the true edge than the yellow line.

Figure 4b shows the statistical result over multiple search lines in a single frame. We measure pixel gradient magnitude along fixed length horizontal search lines (the cyan lines in Figure 4a) and then plot them against image *u* coordinates in Figure 4b. The red predicted line is closer to the maxima whereas there is an offset in the yellow line. This offset varies with motion of the camera. More details and experimental results for the line tracker and prediction is presented in [18].

Although we implemented enhancements such as sub-pixel interpolation and feature prediction to improve tracking performance, the line tracker still suffered (tracking failures and noisy tracking) in both indoor and outdoor environments for a variety of reasons. In some cases, man-made structures caused tracking of the pole edges to fail because of other strong vertical edge features in the scene. In other cases the tracker was still able to track the pole edges but the tracking was noisy due to the background scene complexity (for example because of trees in the background).

This reveals the limitations of a naive gradient magnitude-based tracker. Robust and accurate object detection algorithms can be utilized to address this challenge. The tracker only searches within the region-of-interest (ROI) determined by the algorithms. However, we have to scarify update rates or agility of the flying robot due to onboard computational limits.

## 3. Position Based Visual Servoing (PBVS)

**Figure 5:**
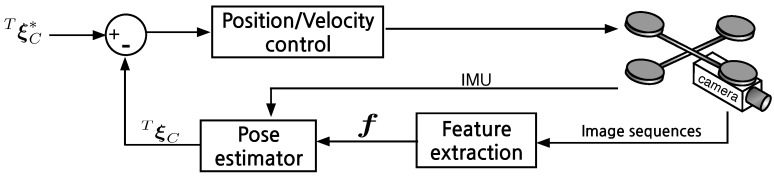
Position-based visual servoing diagram. ***f*** is a feature vector. ${}^{T}\xi_{C}$ and ${}^{T}\xi_{C}^{*}$ are the estimated and the desired pose of the target with respect to the camera.

**Figure 6:**
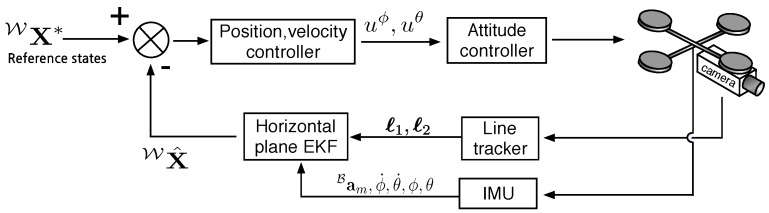
Block diagram of horizontal plane state estimator and control used for the PBVS approach. $u^{\phi }$ and $u^{\theta }$ denote control inputs for roll and pitch commands. $\ell_{1}$ and $\ell_{2}$ are tracked 2D lines and ${}^{B}a_{m}$ is onboard inertial measurement unit (IMU) acceleration measurement.

PBVS uses measured visual features, camera calibration parameters, and prior knowledge about the target in order to determine the pose of a camera with respect to the target. We use an Extended Kalman Filter for state estimation with a Plücker line representation as shown in Figure 5. State estimation and control are performed in SE(3). This section presents details of the horizontal and vertical Kalman Filter frameworks shown in Figure 6 and simulation results.

### 3.1. Horizontal Plane EKF

The position and velocity of the vehicle in the horizontal plane is estimated using monocular vision and inertial data. These sensor modalities are complementary in that the IMU outputs are subject to drift over time, whereas the visually acquired pole edge measurements are drift free and absolute with respect to the world frame, but of unknown scale.

#### 3.1.1. Process Model

Our discrete-time process model for the flying body assumes constant acceleration [33].

(8) $$\begin{array}{c} {}^{W}\hat{X}\langle k+1|k\rangle =A{}^{W}\hat{X}\langle k|k\rangle +Bb_{k}+v \end{array}$$

where ${}^{W}X_{k}={\left[{}^{W}x_{k},{}^{W}y_{k},{}^{W}{\dot{x}}_{k},{}^{W}{\dot{y}}_{k},\phi_{k},\theta_{k}\right]}^{T}$. There is an ambiguity for ${}^{W}y$ and yaw angle (*ψ*), as both result in the target appearing to move horizontally in the image. Although these are the observable states by both the camera and the IMU, it is a challenge to decouple them with our front-facing camera configuration and without using additional sensors. Therefore, we omit yaw (heading) angle estimation in the EKF states and assume it is controlled independently, for example using gyroscope and/or magnetometer sensors.

$\hat{X}\langle k+1|k\rangle$ is the estimate of ***X*** at time k+1 given observations up to time *k*. $b_{k}={\left[{}^{W}{\ddot{x}}_{k},{}^{W}{\ddot{y}}_{k},{\dot{\phi }}_{k},{\dot{\theta }}_{k}\right]}^{T}$ represents the sensor-observed motion of the vehicle. ***A*** and ***B*** describe the evolution of a state vector and are given by

(9) $$\begin{array}{cc} A= & \left[\begin{array}{cccccc} \;1 & \;0 & \;\Delta t & \;0 & \;0 & \;0\\ \;0 & \;1 & \;0 & \;\Delta t & \;0 & \;0\\ \;0 & \;0 & \;1 & \;0 & \;0 & \;0\\ \;0 & \;0 & \;0 & \;1 & \;0 & \;0\\ \;0 & \;0 & \;0 & \;0 & \;1 & \;0\\ \;0 & \;0 & \;0 & \;0 & \;0 & \;1 \end{array}\right],B=\left[\begin{array}{cccc} \;\frac{1}{2}\Delta t^{2} & \;0 & \;0 & \;0\\ \;0 & \;\frac{1}{2}\Delta t^{2} & \;0 & \;0\\ \;\Delta t & \;0 & \;0 & \;0\\ \;0 & \;\Delta t & \;0 & \;0\\ \;0 & \;0 & \;\Delta t & \;0\\ \;0 & \;0 & \;0 & \;\Delta t \end{array}\right] \end{array}$$

It is worth mentioning that accelerometers measure the difference between the actual acceleration of a robot and the gravity vector in {*B*} [34,35]. Therefore, accelerations in {*W*} are

(10) $$\begin{array}{c} {}^{W}a=\left[\begin{array}{c} {}^{W}\ddot{x}\\ {}^{W}\ddot{y}\\ {}^{W}\ddot{z} \end{array}\right]={}^{W}R_{B}{}^{B}a_{m}-g \end{array}$$

where g is gravitational acceleration, ${\left[0,0,g\right]}^{T}$ and ${}^{B}a_{m}$ is the accelerometer measurement. Process noise v is assumed to be Gaussian in nature:

(11) $$\begin{array}{cc} \text{v} & ∼N(0,Q)\\ Q & =\text{diag}\left[\begin{array}{cccccc} \sigma_{{}^{W}x}^{2} & \sigma_{{}^{W}y}^{2} & \sigma_{{}^{W}\dot{x}}^{2} & \sigma_{{}^{W}\dot{y}}^{2} & \sigma_{\phi }^{2} & \sigma_{\theta }^{2} \end{array}\right] \end{array}$$

where Q is the covariance matrix of the process noise. N(0,Q) denotes a zero-mean Gaussian noise process, and *σ* is the standard deviation of the corresponding states. The covariance propagation step follows the standard Kalman Filter procedure.

#### 3.1.2. Measurement Model

**Figure 7:**
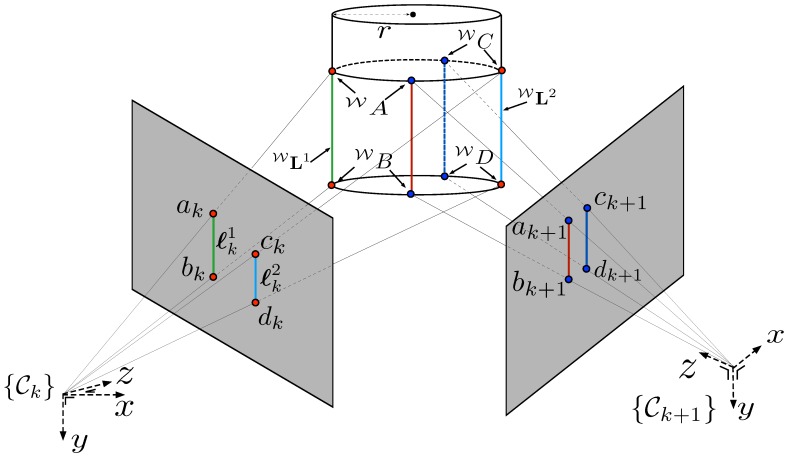
Projection model for a cylindrical object. ${}^{W}A,{}^{W}B,{}^{W}C,{}^{W}D\in R^{3}$ denote points in the world frame with $a_{k},b_{k},c_{k},d_{k}\in R^{2}$ denoting their corresponding projection onto a planar imaging surface at a sample *k*. Although we actually measure different world points between frames, they are considered to be the same point due to the cylindrical nature of the object and the choice of line representation.

Four points $\in R^{3}$ that lie on the two sides of the pole are defined in {W}. Two Plücker lines, ${}^{W}L^{1}$ and ${}^{W}L^{2}$, are formed and projected onto the image plane as shown in Figure 7.

We partition the measurement into two components: visual $z_{\mathrm{cam}}$ and inertial $z_{\mathrm{IMU}}$. The measurement vector is

(12) $$\begin{array}{c} Z_{k}=\left[\begin{array}{c} \ell_{k}^{1}\\ \ell_{k}^{2}\\ \phi_{k}\\ \theta_{k} \end{array}\right]=\left[\begin{array}{c} z_{\mathrm{cam}}\\ z_{\mathrm{IMU}} \end{array}\right] \end{array}$$

where $\ell_{k}^{i}\in R^{2}$ are the 2D line features from the tracker as given by Equation (5). The projected line observation is given by the nonlinear function of Equation (3)

(13) $$\begin{array}{cc} z_{\mathrm{cam}}= & \left[\begin{array}{c} h_{\mathrm{cam}}\left({}^{W}L^{1},{}^{W}{\hat{x}}_{k},{}^{W}{\hat{y}}_{k},{\hat{\phi }}_{k},{\hat{\theta }}_{k},w\right)\\ h_{\mathrm{cam}}\left({}^{W}L^{2},{}^{W}{\hat{x}}_{k},{}^{W}{\hat{y}}_{k},{\hat{\phi }}_{k},{\hat{\theta }}_{k},w\right) \end{array}\right] \end{array}$$

(14) $$\begin{array}{cc} = & \left[\begin{array}{c} C\left({}^{W}{\hat{x}}_{k},{}^{W}{\hat{y}}_{k},{\hat{\phi }}_{k},{\hat{\theta }}_{k}\right){}^{W}L^{1}C{\left({}^{W}{\hat{x}}_{k},{}^{W}{\hat{y}}_{k},{\hat{\phi }}_{k},{\hat{\theta }}_{k}\right)}^{T}\\ C\left({}^{W}{\hat{x}}_{k},{}^{W}{\hat{y}}_{k},{\hat{\phi }}_{k},{\hat{\theta }}_{k}\right){}^{W}L^{2}C{\left({}^{W}{\hat{x}}_{k},{}^{W}{\hat{y}}_{k},{\hat{\phi }}_{k},{\hat{\theta }}_{k}\right)}^{T} \end{array}\right] \end{array}$$

Note that the unobservable states ${}^{W}z_{k}$ and *ψ* are omitted. **w** is the measurement noise with measurement covariance matrix, R

(15) $$\begin{array}{cc} w & ∼N(0,R)\\ R & =\text{diag}\left[\begin{array}{cccccc} \sigma_{\alpha 1}^{2} & \sigma_{\beta 1}^{2} & \sigma_{\alpha 2}^{2} & \sigma_{\beta 2}^{2} & \sigma_{\phi }^{2} & \sigma_{\theta }^{2} \end{array}\right] \end{array}$$

We manually tune these parameters by comparing the filter output with Vicon ground truth. We generated the run-time code for Equation (14) using the MATLAB Symbolic Toolbox and then exporting the C++ code. This model is 19 K lines of source code but computation time is just 6 μs.

The update step for the filter requires linearization of this line model and evaluation of the two Jacobians

(16) $$\begin{array}{cc} H_{x}= & \frac{\partial h_{\mathrm{cam}}}{\partial x}{|}_{\hat{X}\left(k\right)},\;\;H_{w}=\frac{\partial h_{\mathrm{cam}}}{\partial w} \end{array}$$

where $H_{x}$ is a function of state that includes the camera projection model. We again use the MATLAB Symbolic Toolbox and automatic code generation (58 K lines of source code) for $H_{x}$. It takes 30 μs to compute in the C++ implementation with the onboard CPU quad-core ARM Cortex-A9.

The remaining observations are the vehicle attitude, directly measured by the onboard IMU ($z_{\mathrm{IMU}}$) and reported at 100 Hz over a serial link. The linear observation model for the attitude is

(17) $$\begin{array}{cc} z_{\mathrm{IMU}}= & \left[\begin{array}{c} \phi_{k}\\ \theta_{k} \end{array}\right]=H_{\mathrm{IMU}}{}^{W}\hat{X} \end{array}$$

(18) $$\begin{array}{cc} H_{\mathrm{IMU}}= & \left[\begin{array}{cccccc} 0 & 0 & 0 & 1 & 0 & 0\\ 0 & 0 & 0 & 0 & 1 & 0 \end{array}\right] \end{array}$$

The measurements $z_{\mathrm{cam}}$ and $z_{\mathrm{IMU}}$ are available at 60 Hz and 100 Hz respectively. The EKF is synchronous with the 60 Hz vision data and the most recent $z_{\mathrm{IMU}}$ measurement is used for the filter update. Inputs and outputs of the horizontal plane EKF are presented in Figure 5.

#### 3.1.3. Simulation Results

**Figure 8:**
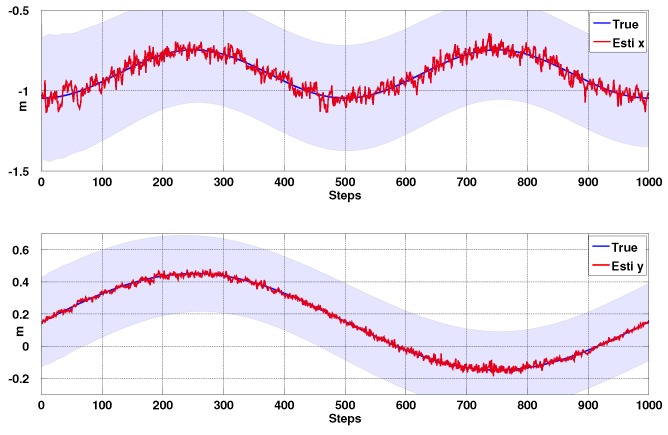
Simulation results for Position Based Visual Servoing (PBVS) tracking: position estimation. ${}^{W}\hat{x}$ and ${}^{W}\hat{y}$ with 3σ confidence boundary.

**Figure 9:**
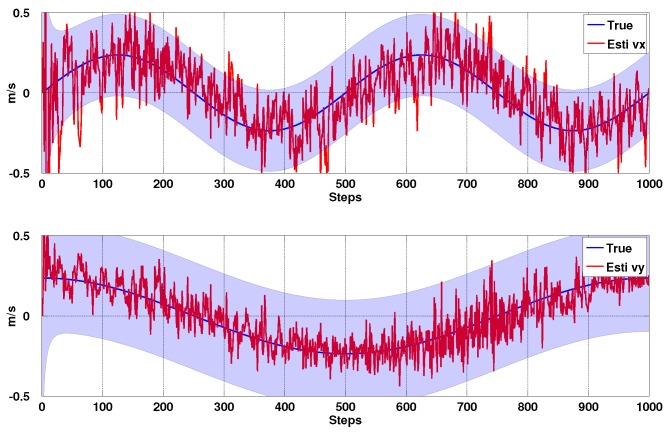
Simulation results for PBVS tracking: velocity estimation. ${}^{W}\hat{\dot{x}}$ and ${}^{W}\hat{\dot{y}}$ with 3σ confidence boundary.

**Figure 10:**
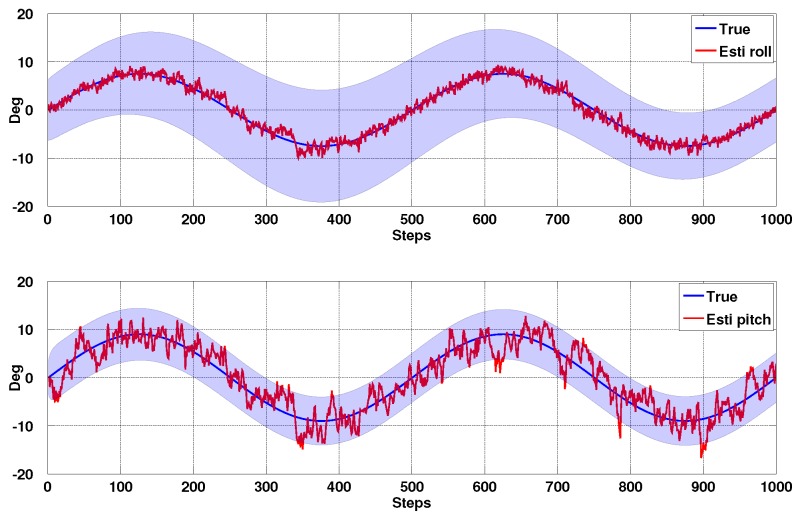
Simulation results for PBVS tracking: angle estimation. $\hat{\phi }$ and $\hat{\theta }$ with 3σ confidence boundary.

In order to validate the EKF line model and Jacobian, we create a virtual camera observing four points, $P_{i}\in R^{3}$ and move the camera with sinusoidal motion in 4 DOF (${}^{W}x,{}^{W}y,\phi ,\theta$) using the simulation framework of [36]. To emulate the errors in line measurements we set the measurement uncertainties to be $\sigma_{\alpha }=1^{\circ }$ and $\sigma_{\beta }=4$ pixels in the line parameters, *α* and *β*, from Equation (5). Estimation results, their confidence boundary and noise parameters are shown in Figure 8, Figure 9 and Figure 10. Most of the states are within 3σ confidence level. The total simulation time is 10 s, with a sampling rate of 100 Hz. We see good quality estimates of position and velocity in the horizontal plane, whilst decoupling the effects of attitude on the projected line parameters. We see that the x-axis forward estimation is noisier than the y-axis since image variation due to change in camera depth is much less than that due to fronto-parallel motion.

### 3.2. Kalman Filter-Based Vertical State Estimation

Various options exist to determine altitude. For instance a downward-looking camera with an object of known scale on the ground and/or vertical visual odometry on the target object (pole) using the forward-facing camera. Due to onboard computational limits we opt, at this stage, to use a sonar altimeter. We observe altitude directly using a downward-facing ultrasonic sensor at 20 Hz, but this update rate is too low for control purposes and any derived velocity signal has too much lag. Therefore we use another Kalman Filter to fuse this with the 100 Hz inertial data which includes vertical acceleration in {B}. The sonar sensor is calibrated by least square fitting to ground truth state estimates. The altitude and z-axis acceleration measurement in {B} are transformed to {W} using $\hat{\phi }$ and $\hat{\theta }$ angles and Equation (10). ${}^{W}X^{\mathrm{alt}}$ is the vertical state, ${\left[{}^{W}z,{}^{W}\dot{z}\right]}^{T}$ and the process model is given by

(19) $$\begin{array}{cc} {}^{W}{\hat{X}}_{\langle k+1|k\rangle }^{\mathrm{alt}}= & A^{\mathrm{alt}}\left[\begin{array}{c} {}^{W}{\hat{z}}_{\langle k|k\rangle }\\ {}^{W}{\hat{\dot{z}}}_{\langle k|k\rangle } \end{array}\right]+B^{\mathrm{alt}}{}^{W}\ddot{z}+v^{\mathrm{alt}} \end{array}$$

where

(20) $$\begin{array}{cc} A^{\mathrm{alt}}= & \left[\begin{array}{cc} 1 & \Delta t\\ 0 & 1 \end{array}\right],\;\;\;B^{\mathrm{alt}}=\left[\begin{array}{c} \frac{1}{2}\Delta t^{2}\\ \Delta t \end{array}\right] \end{array}$$

and where $v^{\mathrm{alt}}$ is the process noise vector of ${}^{W}z$ and ${}^{W}\dot{z}$. The covariance matrices of the process and measurement noise, $Q^{\mathrm{alt}}$ and $R^{\mathrm{alt}}$, are defined as Equations (12) and (16). The observation matrix is $H^{\mathrm{alt}}=\left[\begin{array}{cc} 1 & 0 \end{array}\right]$.

## 4. Image Based Visual Servoing (IBVS)

**Figure 11:**
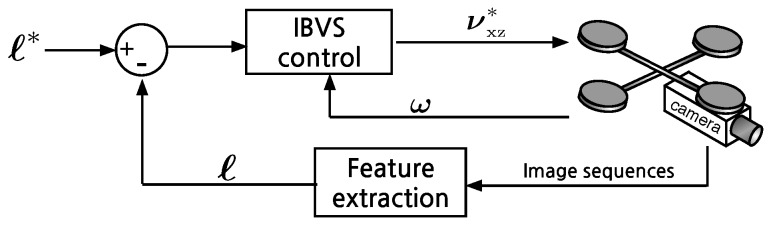
Image-based visual servoing diagram. We model an ordinary camera which has 3mm focal length, 320×240 image resolution. $\nu_{\mathrm{xz}}^{*}$ is the computed desired translational velocity and ***ω*** is used for de-rotation in the Imaged-Based Visual Servoing (IBVS) control block.

Image based visual servoing (IBVS) omits the pose estimation block of Figure 5 and the control is computed directly from image-plane features as shown in Figure 11. It is a challenging control problem since the image features are a non-linear function of camera pose, and the controller generates desired velocities which the non-holonomic platform cannot follow. In this section we present the relation between camera and image motion, the image Jacobian for line features and an IMU-based de-rotation technique. Simulation results are also presented.

### 4.1. Line-Feature-Based IBVS

IBVS has been exploited in a wide range of robotic applications mainly due to its simplicity and robustness to control error [31,37,38]. Point-feature-based IBVS systems are used commonly because point features are fundamental, general and visually distinct in the image. State-of-the-art scale and rotation invariant feature tracking techniques have been used to demonstrate robust and accurate IBVS. By comparison line-feature-based IBVS implementations are relatively rare, yet lines are distinct visual features in man-made environments, for examples the edges of roads, buildings and power distribution poles.

#### 4.1.1. Image Jacobian for Line Features

**Figure 12:**
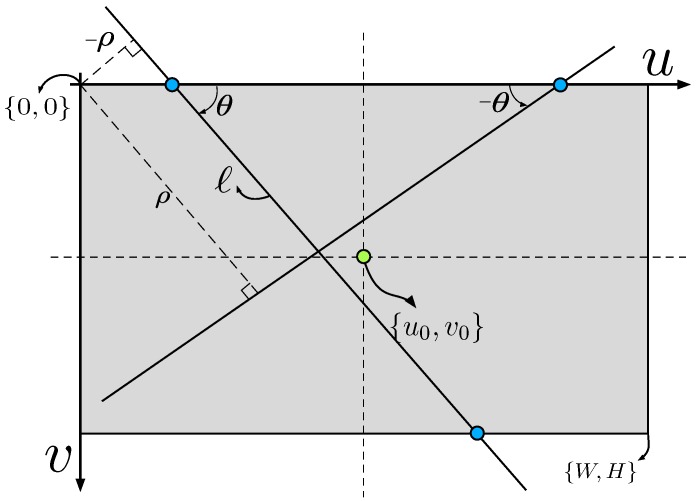
An example of the (*ρ*-*θ*) representation for two lines, *ℓ*. Signs of the two parameters, *ρ* and *θ*, are shown for the corresponding positive or negative quantities. $\left\{u_{0},v_{0}\right\}$ is the principle point and {W,H} denotes the width and the height of the image plane. The origin of the pixel coordinate frame is at the top-left of the image plane by convention.

The homogeneous equation of a 2D line is au+bv+c=0 with coefficients (a,b,c). Although any line can be represented in this form it does not have a minimum number of parameters. The standard *slope-intercept* form v=mu+c where *m* is slope and *c* is intercept is problematic for the case of vertical lines where m=∞. We therefore choose (ρ,θ) parameterization as the 2D line representation as shown in Figure 12

(21) $$\begin{array}{c} u\sin\theta +v\cos\theta =\rho \end{array}$$

where $\theta \in [-\frac{\pi }{2},\frac{\pi }{2})$ is the angle from the *u*-axis to *v*-axis in radians, and $\rho \in [-\rho_{\min},\rho_{\max}]$ is the perpendicular distance in pixels from the origin to the line. This form can represent a horizontal line (θ=0) and a vertical line ($\theta =-\frac{\pi }{2}$).

For a moving camera, the rate of change of line parameters is related to the camera velocity by

(22) $$\dot{\ell }=\left[\begin{array}{c} \dot{\theta }\\ \dot{\rho } \end{array}\right]=J_{l}\nu$$

where $\dot{\theta }$ and $\dot{\rho }$ are the velocity of a line feature, and are analogous to optical flow for a point feature. These line parameters are simply related to the line parameters introduced earlier by

(23) $$\theta =\tan^{-1}\alpha ,\;\;\rho =\beta \cos\theta$$

The matrix $J_{l}$ is the *Image Jacobian* or *Interaction matrix* and given by Equation (29) [39]. The lines lie on the equation of a plane AX+BY+CZ+D=0 where (A,B,C) is the plane normal vector and *D* is the distance between the plane and the camera. The camera spatial velocity in world coordinates is

(24) $$\begin{array}{cc} \nu & ={\left[\begin{array}{c} v_{x},v_{y},v_{z}\;\;|\;\;\omega_{x},\omega_{y},\omega_{z} \end{array}\right]}^{T}\\ & ={\left[\nu_{t},\;,|,\;,\omega \right]}^{T} \end{array}$$

where $\nu_{t}$ and ***ω*** are the translational and angular velocity components respectively.

For the case of *N* line features we can stack these equations. The left hand side is a 2N×1 matrix, while the stacked Jacobian is 2N×6. In this paper, we consider the N=2 case where the two lines are the vertical edges of the pole, which yields

(25) $$\begin{array}{c} \left[\begin{array}{c} {\dot{\theta }}_{1}\\ {\dot{\rho }}_{1}\\ {\dot{\theta }}_{2}\\ {\dot{\rho }}_{2} \end{array}\right]=\left[\begin{array}{c} J_{l_{1}}\\ J_{l_{2}} \end{array}\right]\nu \end{array}$$

We can solve for the camera motion required in order to move the image features to the desired value

(26) $$\begin{array}{c} \nu =J_{l}^{+}\dot{\ell } \end{array}$$

where $J_{l}^{+}$ denotes the pseudo-inverse of $J_{l}$. Given the desired feature vector, $\ell^{*}$, the desired feature velocity is

(27) $$\begin{array}{c} {\dot{\ell }}^{*}=\lambda \left(\ell^{*}-\ell \right) \end{array}$$

where *ℓ* represents the two tracked line features, $\ell^{*}$ is the desired feature positions in the image plane, and *λ* is a positive scalar for a simple linear controller. Substituting Equation (27) into Equation (26) yields the desired camera velocity:

(28) $$\begin{array}{c} \nu^{*}=\lambda J_{l}^{+}\left(\ell^{*}-\ell \right) \end{array}$$

It is important to note that we do require some *a priori* Cartesian knowledge about the scene: the distance from the camera origin to the plane in which the vertical lines lie and the approximate orientation of that plane. This information is encoded in the parameters of the plane which is required to compute the image Jacobian in Equation (29). We know A,B,C because the plane is vertical and orthogonal to the camera x-axis (*A*=0, *B*=0, and *C*=1). Since we are interested in flying close to the target, we choose a reasonable value for *D*. We will discuss this point further in Section 4.2.

(29) $$J_{l}=\left[\begin{array}{cccccc} \lambda_{\theta }\sin\theta & \lambda_{\theta }\cos\theta & -\lambda_{\theta }\rho & -\rho \sin\theta & -\rho \cos\theta & -1\\ \lambda_{\rho }\sin\theta & \lambda_{\rho }\cos\theta & -\lambda_{\rho }\rho & -(1+\rho^{2})\cos\theta & (1+\rho^{2})\sin\theta & 0 \end{array}\right]$$

$$\text{where}\;\;\;\;\;\lambda_{\rho }=\frac{A\rho \sin\theta +B\rho \cos\theta +C}{D},\lambda_{\theta }=\frac{A\cos\theta -B\sin\theta }{D}$$

#### 4.1.2. Unobservable and Ambiguous States with Line Features

Depending on the number of lines and their orientation it may not be possible to recover all camera velocity elements. Some velocities may be unobservable, that is, camera motion in that direction causes no change in the image. Some observed motion may be ambiguous, that is, the same image motion might be caused by two or more different camera velocities. In order to recover all elements of the camera velocity we need to observe at least 3 non-parallel lines. These limitations can be found from examining the null-space of the Jacobian and its dimensions give the number of ambiguous states [40], and are summarised in Table 1. The unobservable velocities could be estimated by alternative sensors such as gyroscopes, magnetometers, or perhaps a downward looking camera that served as a visual compass. These alternative estimates could also be used to resolve ambiguities.

For the case of two vertical lines considered in this paper, the vertical velocity is unobservable and there is ambiguity between a sideways motion (camera x-axis) and a rotation about camera y-axis. Another manifestation is the case where a change in more than one state causes the same feature motions in the image. For example a sideways motion (camera x-axis) and a rotation about camera y-axis.

**Table 1:** Unobservable and ambiguous velocity components.

# of lines	Rank	Unobservable	Ambiguities	Condition
1	2	*v_y_*	*v_x_* ∼ *v_z_* ∼ *ω_y_*, *ω_x_* ∼ *ω_z_*	Line not on the optical axis
2	4	*v_y_*	*v_x_* ∼ *ω_y_*	—
3	6 (Full)	—	Lines are not parallel	

#### 4.1.3. De-Rotation Using an IMU

VTOL platforms such as a quadrotor or a hexarotor are underactuated and cannot translate without first tilting the thrust vector in the direction of travel. This rotation immediately causes the image features to move and increases the image feature error, causing poor performance with a simple linear controller like Equation (28). Instead we use IMU measurement of this rotation which we subtract from the observed feature motions, often called *image de-rotation* [41]. The displacements of line features in *θ* and *ρ* are a function of a camera rotation about the x, y and z axes in the world coordinate: roll, pitch and yaw. We rewrite Equation (25) in partitioned form [37] as

(30) $$\begin{array}{cc} \dot{\ell } & =\left[\begin{array}{c} \frac{1}{D}J_{t}\;\;|\;\;J_{\omega } \end{array}\right]\left[\begin{array}{c} \nu_{xz}\\ \omega \end{array}\right]\\ & =\frac{1}{D}J_{t}\nu_{xz}+J_{\omega }\omega \end{array}$$

where $\dot{\ell }$ is a 4×1 optical flow component, $\frac{1}{D}J_{t}$ and $J_{\omega }$ are 4×2 translational and 4×3 rotational components. They are respectively columns {1,3} and {4,5,6} of the stacked Jacobian, ${\left[\begin{array}{c} J_{l_{1}},J_{l_{2}} \end{array}\right]}^{T}$. Note we omit column {2} which corresponds to the unobservable state, $v_{y}$. The reduced Jacobian is slightly better conditioned (smaller condition number) and the mean computation time is measured at 30 μs which is 20 μs faster than for the full size Jacobian. Thus $\nu_{\mathrm{xz}}$ contains only two elements, ${\left[\begin{array}{c} v_{x},v_{z} \end{array}\right]}^{T}$, the translational camera velocity in the horizontal plane. This is input to the vehicle’s roll and pitch angle control loops. The common denominator, *D*, denotes target object depth which is assumed, and *ω* is obtained from an IMU. We rearrange Equation (30) as

(31) $$\begin{array}{c} \nu_{\mathrm{xz}}=DJ_{t}^{+}\left(\dot{\ell }-J_{\omega }\omega \right) \end{array}$$

and we substitute Equation (27) into Equation (31) to write

(32) $$\begin{array}{c} \nu_{\mathrm{xz}}^{*}=DJ_{t}^{+}\left(\lambda \left(\ell^{*}-\ell \right)-\underset{︸}{J_{\omega }\omega }\right) \end{array}$$

The de-rotation term is indicated, and subtracts the effect of camera rotation from the observed features. After subtraction, only the desired translational velocity remains.

**Figure 13:**
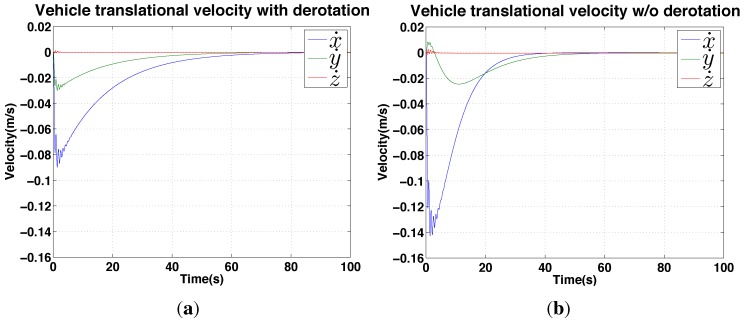
Simulation results for IBVS regulation: velocity response. (**a**) with de-rotation; (**b**) without.

### 4.2. IBVS Simulation Results

In order to validate the IBVS system and de-rotation method, we use a simulation framework from [36] for a quadrotor dynamic model equipped with a front-facing perspective camera. We implement the mentioned IBVS system with two vertical lines and the de-rotation block in the IBVS control block which yields the required translational velocity, $\nu_{\mathrm{xz}}^{*}$.

**Figure 14:**
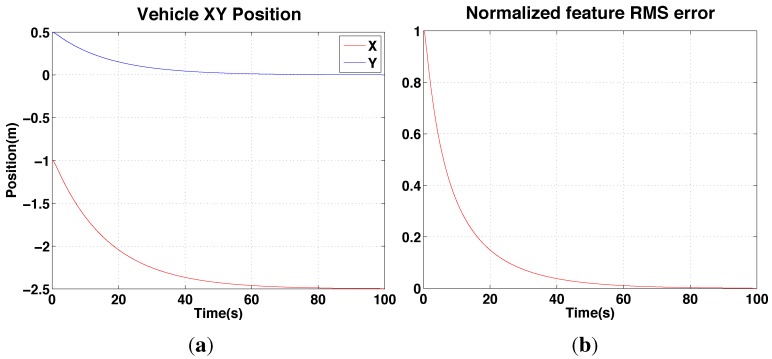
Simulation results for IBVS (with de-rotation): position response. (**a**): vehicle position (x,y) *versus* time showing the vehicle achieves its goal (x = −2.5, y = 0) at around 60 s; (**b**) the normalized root mean squared feature error ($\ell^{*}-\ell$) for the same period.

The vehicle is initially located at (x,y) = (−1.0, 0.5) m and the goal position is (−2.5, 0) m in the world coordinate frame. Figure 14 shows the results of the position changes in *x* and *y* and the normalized feature errors over time. There are two parameters, λ=0.3 and D=1 which are respectively a positive gain and a depth for this simulation.

We ran simulations with and without the de-rotation block for comparison. Although the vehicle converges to the goal position slower with de-rotation enabled (≈38s *versus* ≈60s) as shown in Figure 13, the de-rotation yields smoother velocity commands and less oscillation of the y velocity.

Computing Equation (29) requires knowledge of the plane normal vector and *D*, the distance from the camera image plane to the plane on which the lines lie [42]. In the literature, many approaches have been demonstrated for depth estimation, e.g., 3D reconstruction of a scene using vision techniques [43], a Jacobian matrix estimator using the kinematics of an arm-type robot and image motion [44] or an Unscented Kalman Filter based estimator using point features and inertial sensor data [45]. These approaches are applicable to our problem however we lack sufficient computational power on our onboard computer to implement them in real time.

Instead we used a fixed value of D (denoted D${}_{\text{fixed}}$), and set this to a value that is reasonable for the pole inspection task (for example 1.5 m). IBVS uses D${}_{\text{fixed}}$ in Jacobian calculation Equation (29) and it is neither the true depth nor current camera pose.

**Figure 15:**
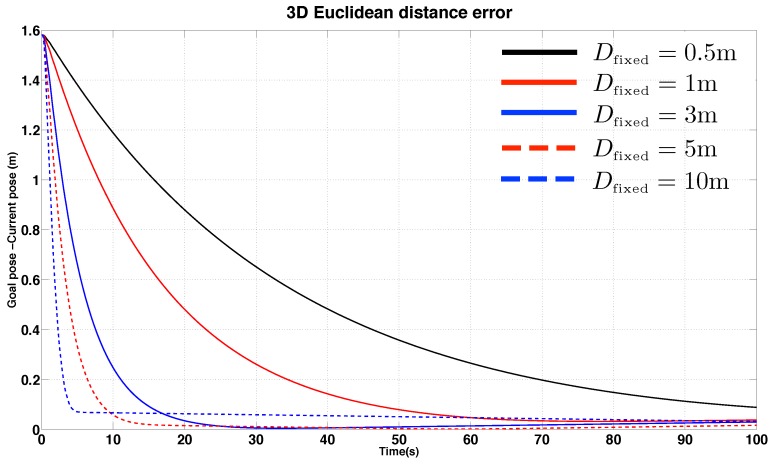
Simulation results for IBVS regulation: error response as a function of time for different assumed constant target object depths.

To investigate the control sensitivity to incorrect values of D${}_{\text{fixed}}$, we ran simulations with different values of D${}_{\text{fixed}}$ and the results are plotted in Figure 15. The figure shows how the camera Euclidean distance error (in SE(3), between the goal and current position) changes over time for a variety of values of D${}_{\text{fixed}}$. The true value of D at t = 0 was 1.5 m. The plot shows that D${}_{\text{fixed}}$ effectively acts as a proportional gain, with higher values of D${}_{\text{fixed}}$ causing faster convergence (for example when the true value of D is 1.5 m, a value of D${}_{\text{fixed}}$ = 5 m results in convergence after 10s compared to 70s for D${}_{\text{fixed}}$ = 1 m). Since D${}_{\text{fixed}}$ acts as a proportional gain, there is the potential for the system to become unstable if the difference between D${}_{\text{fixed}}$ and D is too large. Figure 15, however, shows that the system is stable for a relatively large variation in D${}_{\text{fixed}}$ values, indicating that using a fixed value for D instead of estimating it online is appropriate for our application.

## 5. Shared Autonomy

A typical attitude-stabilized multi-rotor has four user-controllable degrees of freedom (DOF), namely horizontal position (x,y), height (z), and heading (*ψ*). These are usually controlled indirectly with joysticks where the stick positions are mapped to rates (e.g., the “throttle” stick position is mapped to climb rate), or to angular velocity in the case of yaw. These commands are in the body coordinate frame, making it hard for an operator to control position in the 3-dimensional Cartesian world coordinate frame.

**Figure 16:**
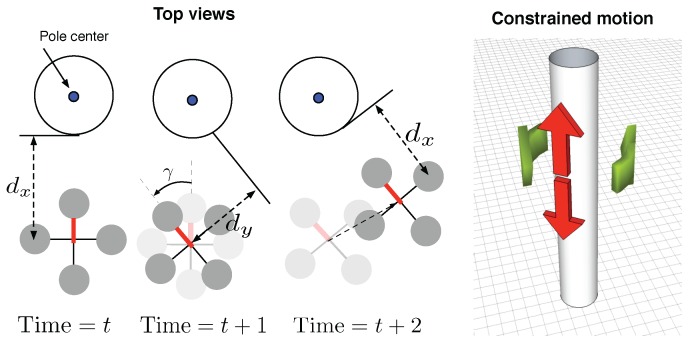
(**left**) illustration of how vehicle induces yaw motion. *γ* is an angle for the yaw motion and $d_{x}$ and $d_{y}$ are distances between the pole and the robot in x-, and y-axis; (**right**) reduced dimension task space for operator commands which is sufficient for inspection purposes.

We propose reducing the operator’s cognitive load and level of skill required by reducing the DOFs that the operator must control, see Figure 16, and letting the system control the remaining DOFs automatically. Additionally, some of the DOFs are controlled in a more direct, intuitive manner rather than indirectly via rate or velocity commands. Since the proposed system can self-regulate the stand-off distance and keep the camera pointed at the target, the operator is left with only two DOFs to control, namely height and yaw rate. Height control is simplified: from the operator providing rate control commands to providing height set-points.

Yaw rate commands are used to induce a translation around the pole, allowing the operator to inspect it from different angles. Changing yaw angle makes the quadcopter circle around the pole (red bar indicates the *front* rotor). References for the x and y position controllers are $d_{x}$ and 0 respectively. The robot hovers by keeping $d_{x}$ distance at time *t*. The operator sends a yaw command and the vehicle rotates by the angle *γ* which induces a lateral offset $d_{y}$ at time t+1. The vision-based controller moves the robot to the right to eliminate $d_{y}$ and keeps $d_{x}$ distance at time t+2—the result is motion around the target object.

## 6. Experimental Results

**Figure 17:**
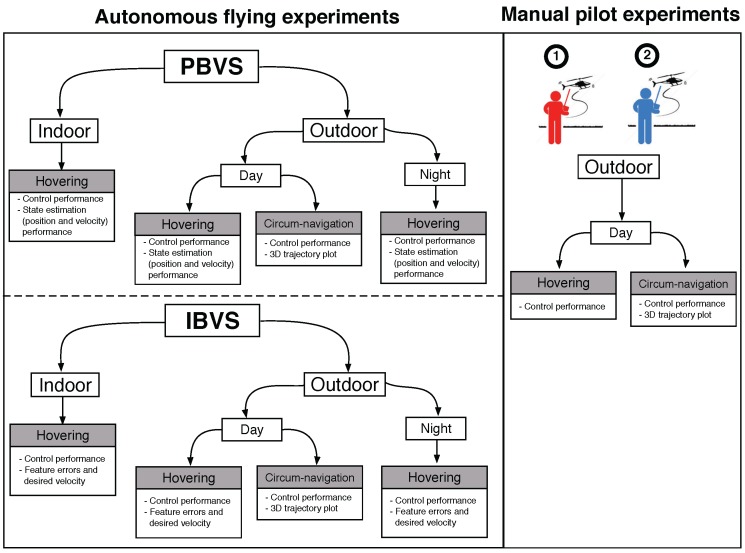
Overview of experiments. There are two categories: autonomous flying with shared autonomy (**left**) and manual piloting with only attitude stabilization (**right**); Autonomous flying consists of PBVS (**top**) and IBVS (**bottom**). Two pilots were involved in the manual piloting experiments. Each box with grey denotes a sub-experiment and describes key characteristics of that experiment.

**Figure 18:**
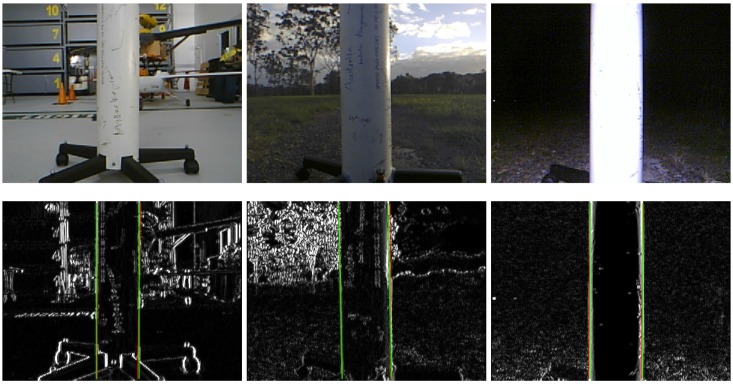
Sample onboard images from the three hovering test environments: indoor (**left**), day-time outdoor (**mid**) and night-time outdoor (**right**) respectively. The top row contains the raw images while the bottom row contains the corresponding gradient images with overlaid lines corresponding to the goal line positions (green) and the tracked pole edges (red).

The experiments we present are summarised in Figure 17 and can be considered with respect to many categories: autonomous or manual, PBVS or IBVS control, hovering or circumnavigation, indoor or outdoor, day or night. The manual pilot experiments pit two human pilots, with different skill levels, against the autonomous system for the tasks of hovering and circumnavigation. Figure 18 shows some sample images from the onboard front-camera captured during various experiments. The demonstration video is available from the following link [22].

### 6.1. Hardware Configuration

Our research hexarotor platform, an Ascending Technologies Firefly, is fitted with a front-facing camera and a quad-core 1.7 GHz ARM Cortex-A9 computer which performs all the processing onboard. It runs Ubuntu Linux, and the Robot Operating System (ROS) [46] is used as the underlying software framework.

The front-facing camera is a low-cost high-speed Playstation EyeToy connected via USB. This CMOS camera has a rolling shutter which is problematic on a moving platform [47]. We thus set essential camera options (such as selecting low-resolution 320 × 240 images, using fixed exposure and gain and the fastest frame rate available) in order to minimize rolling shutter effects. The IMU provides angular velocity as well as orientation (roll, pitch, yaw) and the 3-axis acceleration through the High-Level-Processor (HLP) and Low-Level-Processor (LLP) of the hexarotor platform [48]. Altitude measurements are obtained from a downward-facing ultrasonic sensor. For night time flying a high-powered LED is mounted to the front to illuminate the scene for the onboard camera.

**Figure 19:**
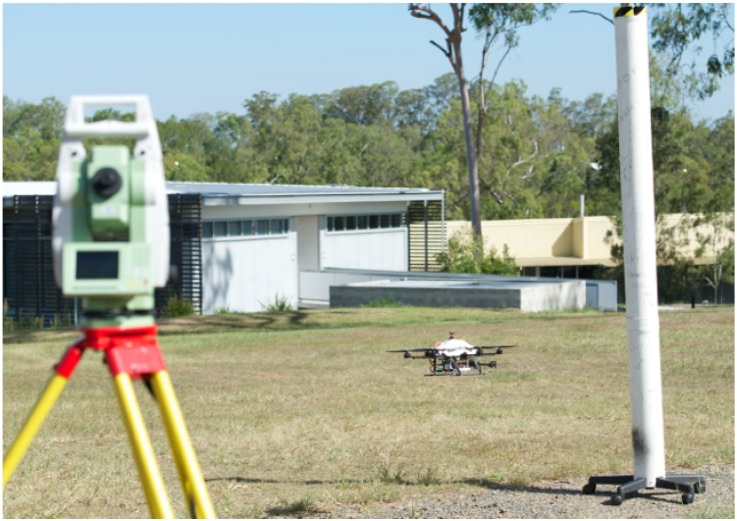
Experimental setup for outdoor flights. The VTOL platform is shown pointing towards the pole. An actuated surveying laser tracks a reflective beacon on the aircraft for position ground truth.

All our experiments are ground truthed. The indoor flights are conducted inside a Vicon motion capture environment and we attach reflective markers to the vehicle which allows us to measure position and attitude at 100 Hz. Outdoors we attach a single corner reflector and use a Leica TS30 laser tracking theodolite which provides position only measurments at 5 Hz shown in Figure 19. Each ground truth system can provide position measurements with sub-millimeter accuracy [49]. We take considerable care to synchronise the clocks of the flying vehicle and the tracking systems to avoid time skew when comparing onboard estimates with ground truth.

#### Software Configuration

**Figure 20:**
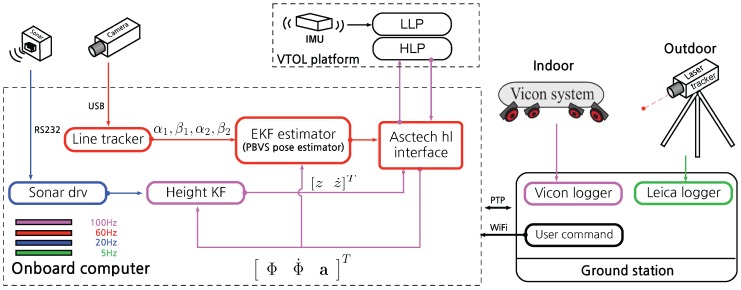
Sensor and software system diagram for PBVS experiments. Different colors denote different sampling rates. The software is implemented using ROS and all computation for a full update cycle happens within 16.6 ms (60 Hz) on average. The line tracker utilizes 55% of the CPU. The Extended Kalman Filter (EKF) includes line model and Jacobian calculations and these steps take 6 μs and 30 μs respectively. Note that only one ground truth source is used at time. The Vicon system is used for indoor ground truth while the Leica laser tracker is used outdoors.

**Figure 21:**
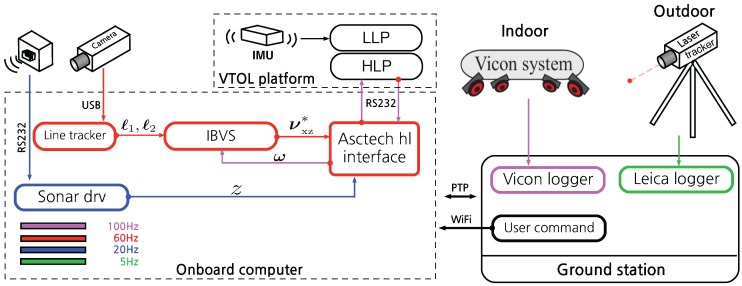
Sensor and software system diagram for IBVS experiments. Different colors denote different sampling rates. All software is implemented using a Robot Operating System (ROS). Note that only one ground truth source is used at time. The Vicon system is used for indoor ground truth while the Leica laser tracker is used outdoors.

We implemented PBVS and IBVS using ROS and Figure 20 and Figure 21 show the sensor and software configurations for PBVS and IBVS control respectively. Different colors denote different sampling rates and arrows denote data flow from the sensors to the software components where processing occurs. Each box is an individual ROS node implemented using C++. Precision Time Protocol (PTP) is utilized for time synchronization between the onboard computer and the external ground truth data logger.

### 6.2. Position-Based Visual Servoing (PBVS)

**Figure 22:**
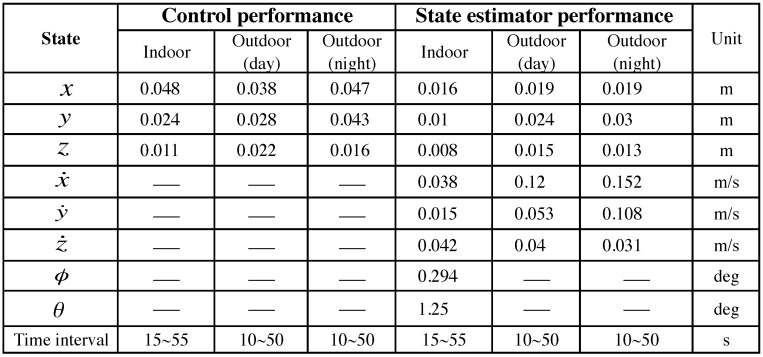
PBVS hovering (standard deviation).

In this section, hovering and circumnavigation experimental results are presented with 4 sub-experiments. During the hovering experiments, no human inputs are provided. Only yaw rate commands are sent to the flying robot for the circumnavigation experiments. We evaluate control performance by comparing the goal and ground truth position. The evaluation of state estimation is presented with ground truth states and onboard estimator outputs. Figure 22 summarizes the position control and state estimation performance for the indoor and outdoor environments.

#### 6.2.1. PBVS Indoor Hovering

**Figure 23:**
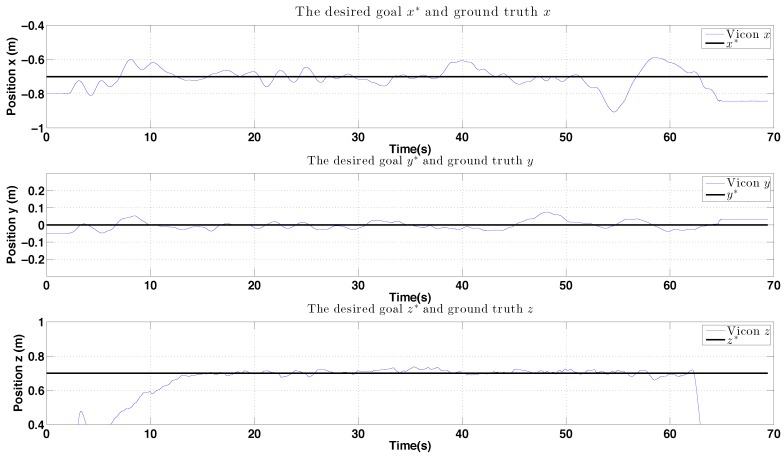
Experimental results for PBVS-based indoor hovering: control performance. Goal (black) and ground truth states (blue). The desired position for ${}^{W}\hat{x}$, ${}^{W}\hat{y}$ and ${}^{W}\hat{z}$ is −0.7 m, 0 m and 0.7 m. We compute standard deviation of errors for each state over the interval 15 s∼63 s: $\sigma_{x}$ = 0.048 m, $\sigma_{y}$ = 0.024 m, and $\sigma_{z}$ = 0.011 m.

The control performance is evaluated by computing the standard deviation of the error between the goal position (x, y and z) and ground truth as measured by the Vicon system. The performance of the controller is shown in Figure 23. Interestingly, although the x-axis velocity estimation is noisy, the control performance for this axis is not significantly worse than for the y-axis, the quadrotor plant is effectively filtering out this noise.

**Figure 24:**
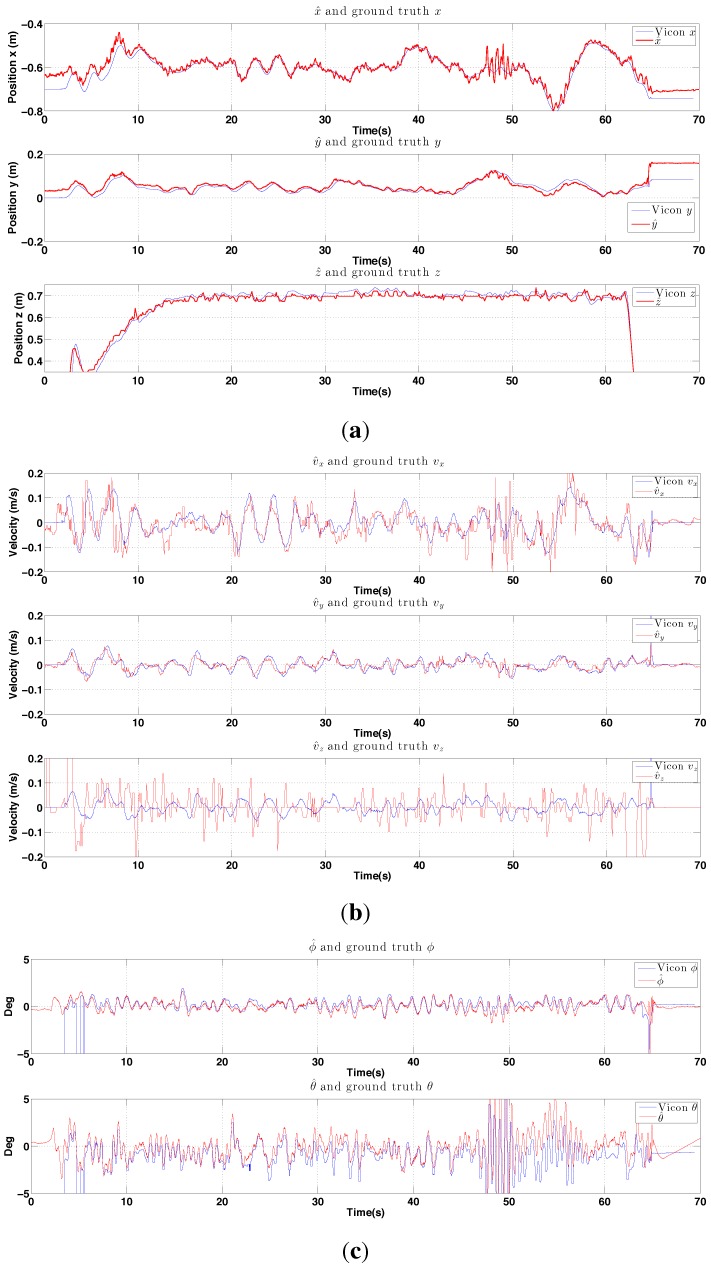
Experimental results for indoor PBVS-based hovering. All states are shown at the same scale. The performance evaluation of the state estimator is summarised in Figure 22. (**a**) Position estimation (red) with ground truth (blue). Note that z-axis is inverted for visualization; (**b**) Velocity estimation (red) with ground truth (blue), ${}^{W}\hat{\dot{x}}$, ${}^{W}\hat{\dot{y}}$ and ${}^{W}\hat{\dot{z}}$; (**c**) Attitude estimation (red) with ground truth (blue), roll $\hat{\phi }$ and pitch $\hat{\theta }$.

We estimate position and orientation except heading angle as shown in Figure 24. The robot oscillates around 48 s–50 s when the line tracker was affected by the noisy background leading to errors in the position estimate ${}^{W}\hat{x}$.

**Figure 25:**
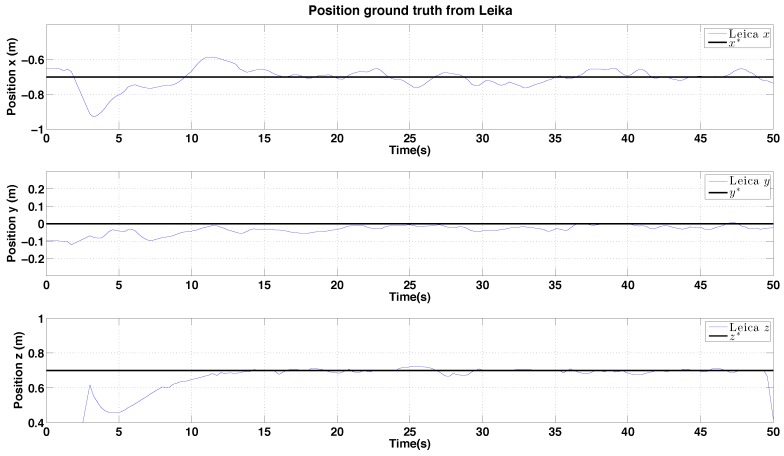
Experimental results for outdoor (day) PBVS-based hovering: control performance. Goal (black) and ground truth states (blue) . The desired position for ${}^{W}\hat{x}$, ${}^{W}\hat{y}$ and ${}^{W}\hat{z}$ is −0.7 m, 0 m and 0.7 m. We compute standard deviations of errors for each state over the interval 10 s∼50 s: $\sigma_{x}$ = 0.038 m, $\sigma_{y}$ = 0.028 m, and $\sigma_{z}$ = 0.022 m.

#### 6.2.2. PBVS Day-Time Outdoor Hovering

**Figure 26:**
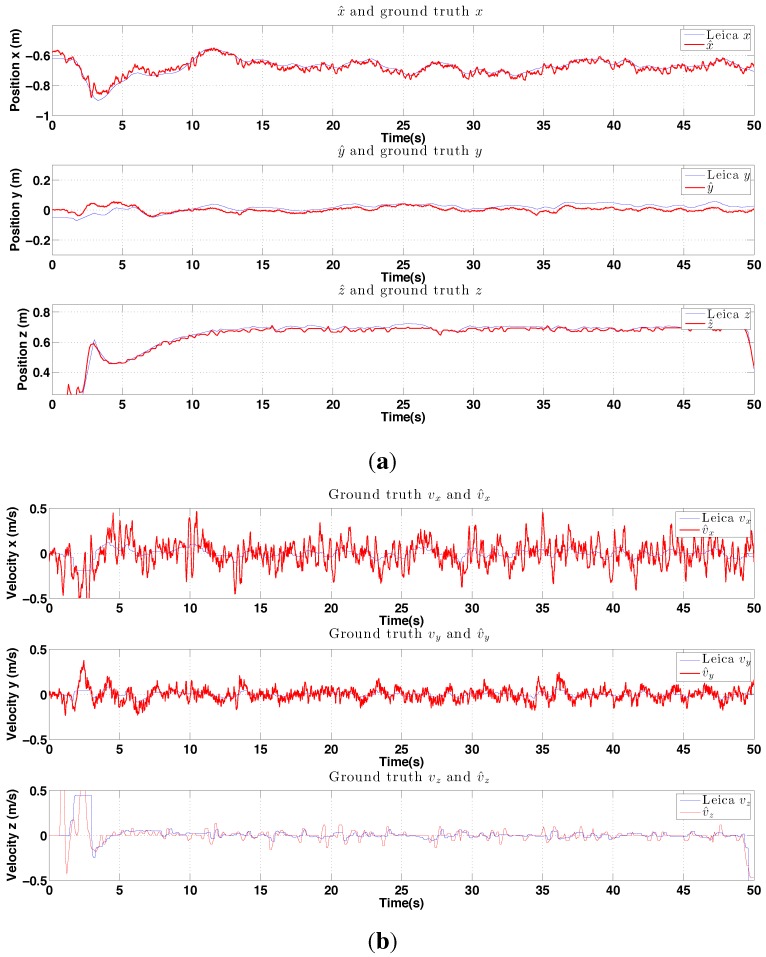
Experimental results for outdoor (day) PBVS-based hovering: estimator performance. Angle estimation are omitted because the laser tracker can only provide position ground truth of the moving target. All states are shown at the same scale. The performance evaluation is presented in Figure 22. (**a**) Position estimation (red) with ground truth (blue). Note that z-axis is inverted for visualization; (**b**) Velocity estimation (red) with ground truth (blue), ${}^{W}\hat{\dot{x}}$, ${}^{W}\hat{\dot{y}}$ and ${}^{W}\hat{\dot{z}}$.

The VTOL platform was flown outdoors where external disturbances such as wind gusts are encountered. In addition, background scenes were nosier as shown in Figure 18. Figure 25 and Figure 26 show control performance and state estimation during day-time hovering outdoors. The proposed system was able to efficiently reject disturbances and maintain a fixed stand-off distance from a pole (see accompanying video demonstration 2.2). Position and velocity estimation results are shown in Figure 26 and are noisier than for the indoor case due to more complex naturally textured background scenes (see Figure 18). Controller performance is consistent with that observed indoors. All results are within a ±0.02 m variation boundary.

#### 6.2.3. PBVS Night-Time Outdoor Hovering

**Figure 27:**
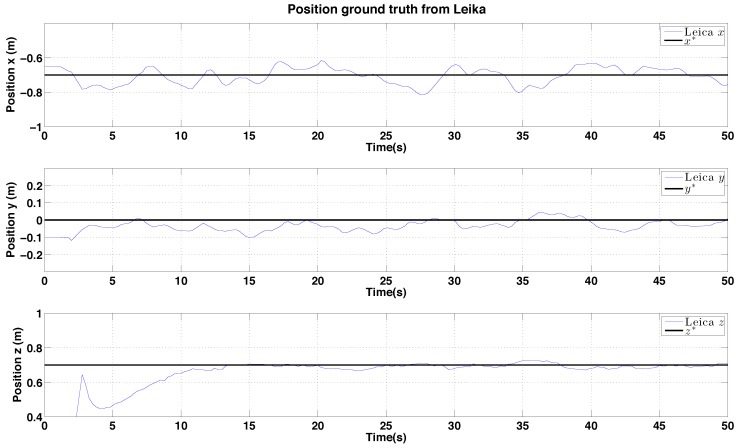
Experimental results for outdoor (night) PBVS-based hovering: control performance. Goal (black) and ground truth states (blue). The desired position for ${}^{W}\hat{x}$, ${}^{W}\hat{y}$ and ${}^{W}\hat{z}$ is −0.7 m, 0 m and 0.7 m. We compute standard deviations of errors for each state over the interval 10 s∼50 s. $\sigma_{x}$ = 0.047 m, $\sigma_{y}$ = 0.043 m, and $\sigma_{z}$ = 0.016 m.

**Figure 28:**
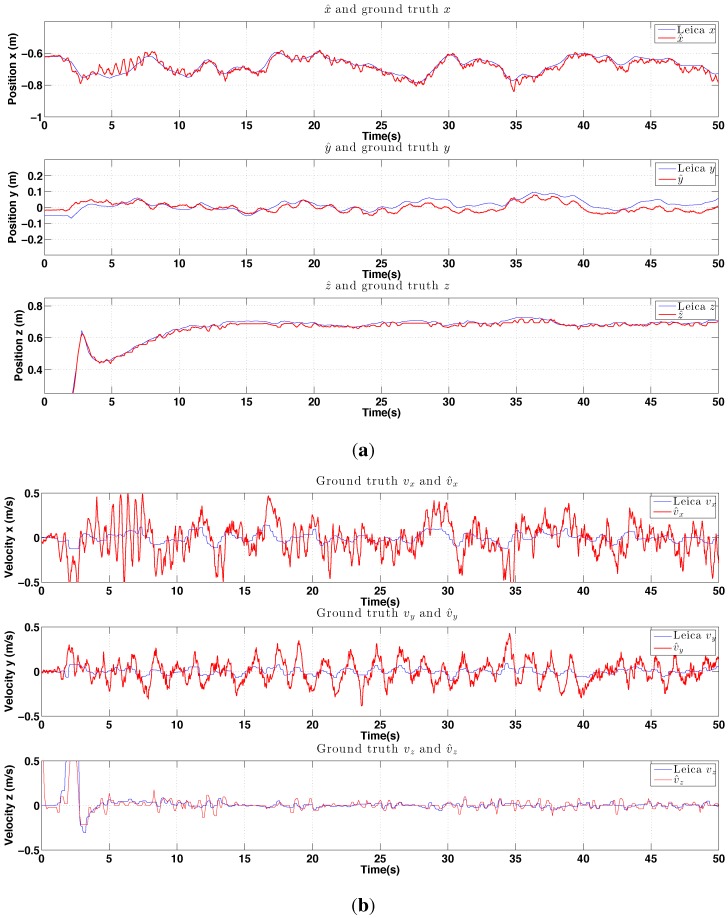
Experimental results for outdoor (night) PBVS-based hovering: estimator performance. Estimated angles are omitted and all states are shown at the same scale. The performance evaluation summary of these plots is presented in Figure 22. (**a**) Position estimation (red) with ground truth (blue). Note that z-axis is inverted for visualization; (**b**) Velocity estimation (red) with ground truth (blue), ${}^{W}\hat{\dot{x}}$, ${}^{W}\hat{\dot{y}}$ and ${}^{W}\hat{\dot{z}}$.

We performed night-time PBVS-based hovering experiments and experienced the best control and state estimation performance shown in Figure 27 and Figure 28 respectively. At night there was less wind (average wind speed was less than 1 m/s) and the pole edges were clear in the image since only the pole in the foreground was illuminated by the onboard light. However, the EKF used for horizontal state estimation was extremely sensitive to the measurement noise parameters, R from Equation (16). We didn’t adapt R for night-time flights (same values as for day-time outdoor and indoor hovering) and this led to poor state estimation and oscillation in the x and y-axes—the worst results among the 3 experiments. This is a potential limitation of the deterministic Extended Kalman Filter (Filter Tuning). [50,51] exploited stochastic gradient descent in order to learn R with accurate ground truth such as motion capture or high quality GPS. We are interested in this adaptive online learning for filter frameworks; however, this is beyond the scope of this work.

#### 6.2.4. PBVS Day-Time Outdoor Circumnavigation

**Figure 29:**
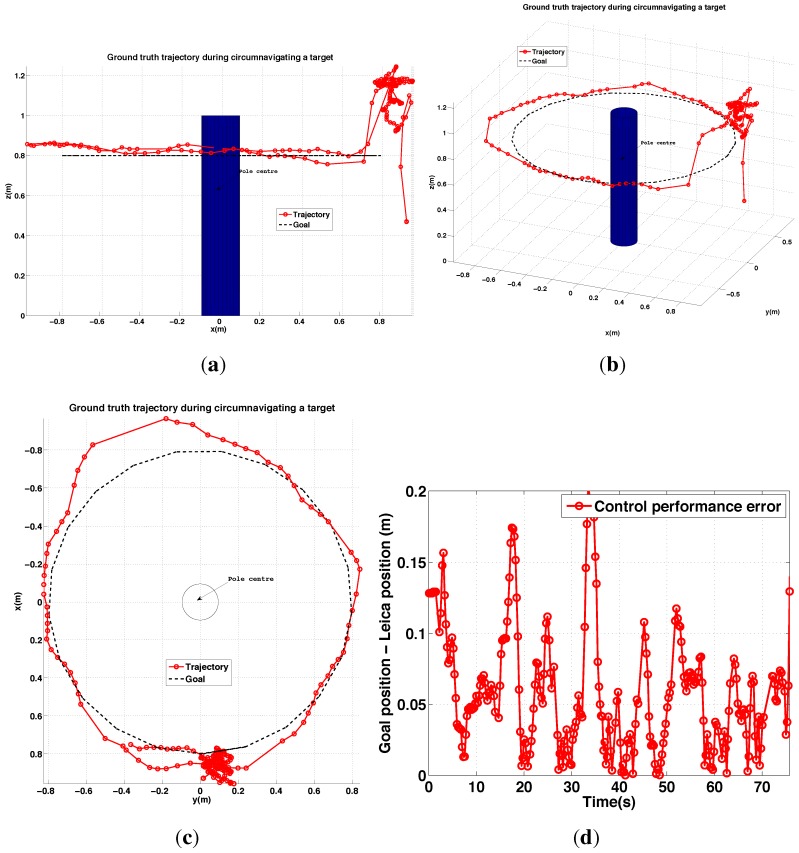
Experimental results for outdoor (day) PBVS-based circumnavigation: control performance. (**a**–**c**) 3D views of the trajectory: Side view, Perspective view and Top view; (**d**) Euclidean error (goal minus actual) trajectory *versus* time.

The pole circumnavigation experiment is performed by placing the VTOL platform on the ground with the camera facing the pole to be inspected and at the desired stand-off distance. The line-tracking algorithm is initialized and the operator then commands only goal height and yaw rate to move the VTOL platform around the pole at different heights. The system keeps the camera oriented towards the pole and maintains the stand-off distance. Figure 29 displays different views of the trajectory for a flight where the pole was circumnavigated. A circle with the goal radius is shown with a dashed line, and we see the system tracks the desired stand-off distance well. At the time the average wind speed was about 1.8 m/s blowing from left to right (See the demonstration video 2.4). The trajectory was within ±0.15 m of the goal radius for most of the circumnavigation but the error increased at around x = −0.7 and y = −0.4 due to wind gusts. Note that the laser tracker lost track of the reflective prism on the vehicle when it was occluded by the pole at (x = −0.8∼−0.9) and (y = −0.6∼−0.2). We computed the standard deviation of the control performance for the flight period (0–75 s) to be 0.034 m. The height change near (x = −0.9, y = 0) is due to the box that was placed on the ground as a takeoff and landing platform. Since the aircraft maintains a fixed height above the ground beneath it, it climbs when flying over the box.

### 6.3. Imaged-Based Visual Servoing (IBVS)

**Figure 30:**
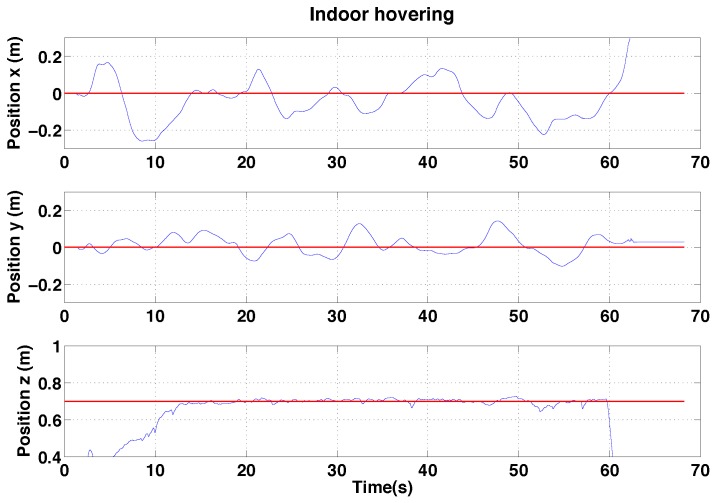
Experimental results for IBVS-based hovering: control performance for indoor IBVS-based hovering. Goal positions shown in red.

**Figure 31:**
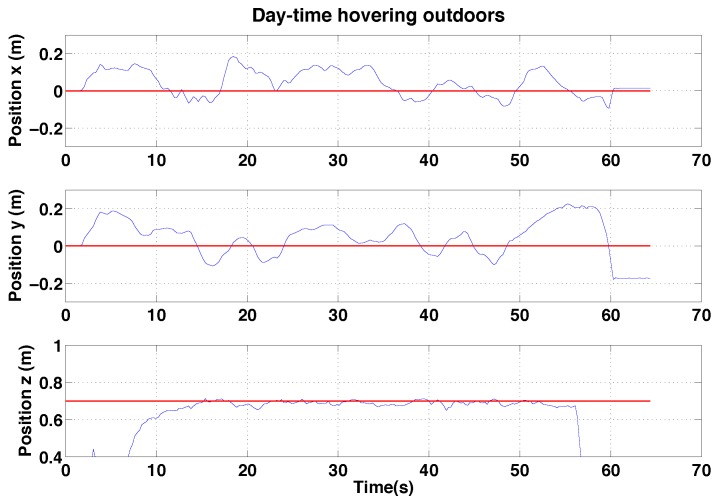
Experimental results for IBVS-based hovering: control performance for outdoor day-time IBVS-based hovering. Goal positions shown in red.

**Figure 32:**
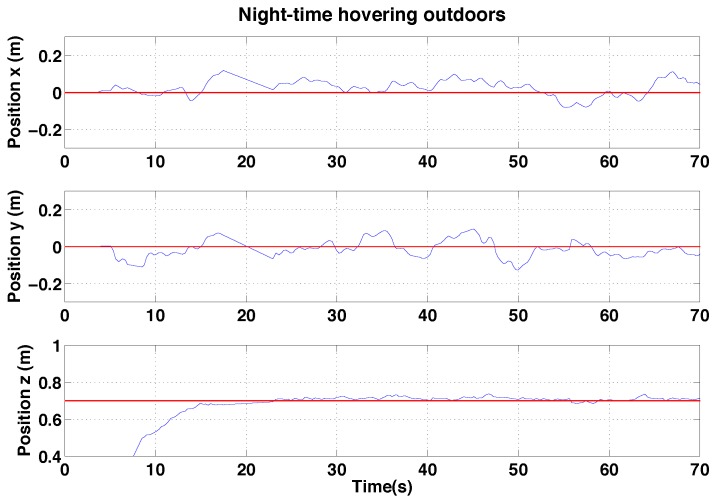
Experimental results for IBVS-based hovering: control performance for night-time IBVS-based hovering. Goal positions shown in red.

We performed pole-relative hovering in 3 environments as shown in Figure 18: indoor (controlled lighting), day-time outdoor and night-time outdoor. For each flight test the platform was flown for approximately a minute and no human interventions were provided during the flight. We set *λ* and *D* to 1.1 and 0.8 m respectively for all experiments presented in this and the following section. A summary of the results is presented in the Table 2, while Figure 30, Figure 31 and Figure 32 show the position results with respect to {W}.

**Table 2:** Hovering standard deviation performance summary of Figure 30, Figure 31 and Figure 32.

State w.r.t { *W*}	Indoor	Outdoor (Day)	Outdoor (Night)	Unit
x	0.084	0.068	0.033	m
y	0.057	0.076	0.050	m
z	0.013	0.013	0.013	m
Duration	15∼60	15∼55	15∼70	s
Wind speed	—	1.8∼2.5	less than 1	m/s

As shown in the Table 2, hovering performance for indoors is similar to that for outdoors despite the fact that there are no wind disturbances indoors. This can be explained by the fact that the hexarotor platform uses a yaw angle estimated from a gyro and magnetometer. Gyros are subject to drift due to biases and vibration noise, while magnetometers are strongly influenced by magnetic perturbations produced by man-made structures indoors. Poor yaw estimates indoors therefore yields a yaw rotation of the vehicle, which in turn causes a y-axis controller error in {*W*}. The platform moves in the body y-axis in order to keep the camera oriented towards the target, and this maneuver also causes an x-axis controller error in practice. Furthermore, salient vertical edges in the man-made indoor environment affect hovering performance as well.

#### 6.3.1. IBVS-Based Hovering

**Figure 33:**
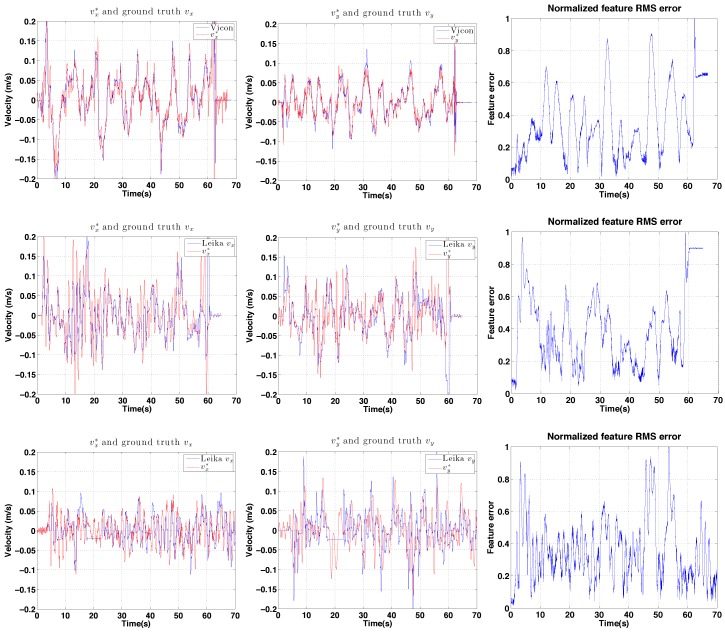
Experimental results for IBVS-based hovering: control demand. $v_{x}^{*}$ (**left column**) and $v_{y}^{*}$ (**middle column**) compared to ground truth. The first row is for indoor hovering while the second and the third rows are for day and night-time outdoor hovering. Normalized root mean squared (RMS) image feature errors for each are shown in the (**right column**).

Control performance of the IBVS-based controller is shown in Figure 30, Figure 31 and Figure 32 for indoors, outdoor day-time and outdoor night-time flight. For the day-time outdoor hovering test the average wind speed was 1.8 m/s with gusts of up to 2.5 m/s (See the demonstration video 1.2). The computed velocity demand to the vehicle is shown in in Figure 33.

The yaw estimation is better outdoors but there are wind disturbances. Also, the line features are noisier due to varying light conditions, shadows, and strong gradients from the background which make the edge of the pole weaker. The best performance was achieved for the outdoor night-time flights. Unlike PBVS, IBVS does not require a pose estimator and is therefore not subject to the EKF sensitivity issue described in Section 6.2.3. As for the PBVS night-time flights, there was less wind at night (average wind speed was less than 1 m/s) and the pole edges were well defined in the image since only the pole in the foreground was illuminated by the onboard light.

#### 6.3.2. IBVS Day-Time Outdoor Circumnavigation

**Figure 34:**
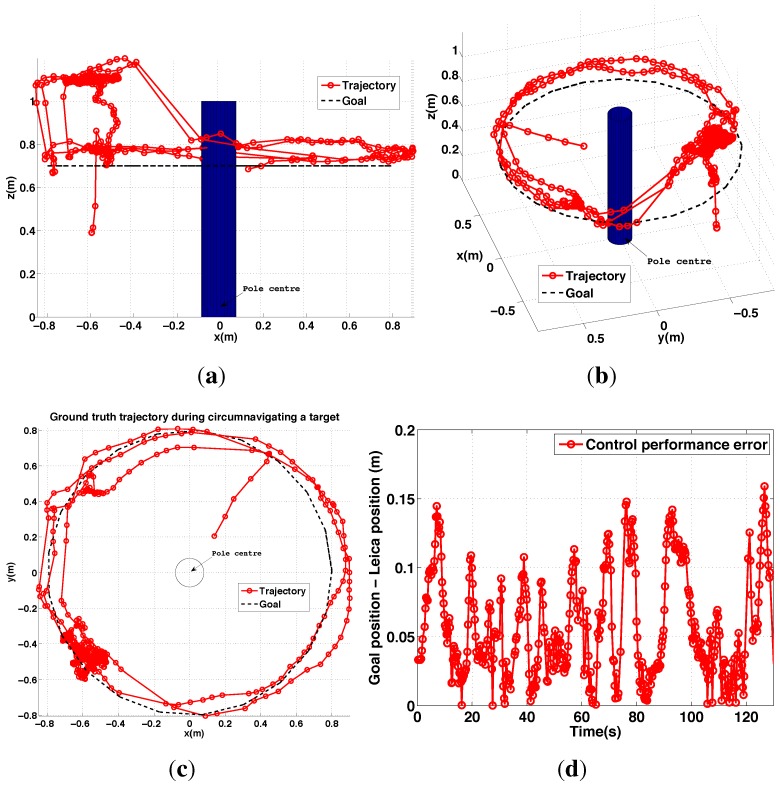
Different views of ground truth trajectory with respect to {*T*} for a pole inspection flight (**a**–**c**); (**d**) is control performance error plot, *i.e.*, 3D Euclidian distance error between the goal and the trajectory. An operator only commands yaw rate using the RC transmitter during the experiment. The constant height, 0.7 m is maintained by the system.

Outdoor circumnavigation experiments were conducted using IBVS in a similar manner to the PBVS experiments. The line-tracking algorithm was initialized and the pole edge features that were found became the desired feature positions for the flight. Figure 34 displays the top, side, and perspective views of the trajectory for a flight where the pole was circumnavigated twice. A circle with the goal radius is shown with a dashed line, and we see the system tracks the desired stand-off distance well. At the time the average wind speed was 1.8 m/s∼2.5 m/s (See the demonstration video 1.4). The stand-off distance was maintained within 0.17 m error boundary for the entire flight as shown in Figure 34d. For comparison with PBVS, We also computed a standard deviation of the control performance error for the same length of flight time (0–75 s) and obtained 0.034 m. IBVS and PBVS show similar performance as shown in Table 3. The height change near (x = −0.6, y = −0.5) is due to the box that was placed on the ground as a takeoff and landing platform. Since the aircraft maintains a fixed height above the ground beneath it, it climbs when flying over the box.

### 6.4. Manually Piloted Experiments

The aim of these experiments was to determine how well a human pilot could perform the inspection tasks, hovering and circumnavigation, that we have demonstrated autonomously. Manual piloting requires great skill and people with this skill are quite scarce. The key skill is hand eye coordination, adjusting the vehicle’s position by controlling roll and pitch angle joysticks. These joysticks effectively control vehicle acceleration which is more difficult for humans to master than the more common rate, or velocity, control inputs. The controls are effected with respect to the vehicle’s coordinate frame, and pilots of moderate skill level are only able to fly with a constant vehicle heading angle, typically the x-axis away from the pilot. Circumnavigation requires the heading angle to change continuously and this requires high-order piloting skills.

**Table 3:** Circum-navigation performance comparison.

	PBVS	IBVS	Unit
Max error margin	0.024	0.017	m
Standard deviation	0.038	0.034	m
Duration	0∼75	0∼125	s

In these experiments we use two pilots with differing skill levels. Pilot 1, one of the authors, has strong skills for manual hovering but is unable to achieve circumnavigation. Pilot 2 is a licensed professional UAV pilot who is able to perform manual circumnavigation flights. In both cases the pilot is making use of the builtin attitude stabilization capability of the vehicle, and the manually piloted experiments were conducted outdoors during the daytime.

**Figure 35:**
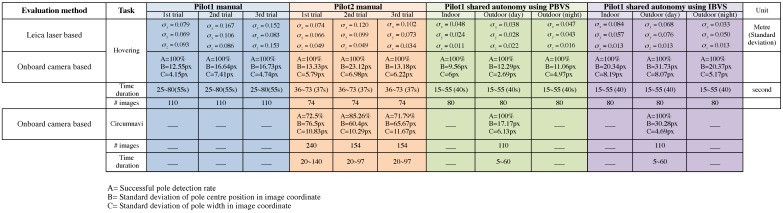
Performance evaluation of manual pilot and autonomous flights.

**Figure 36:**
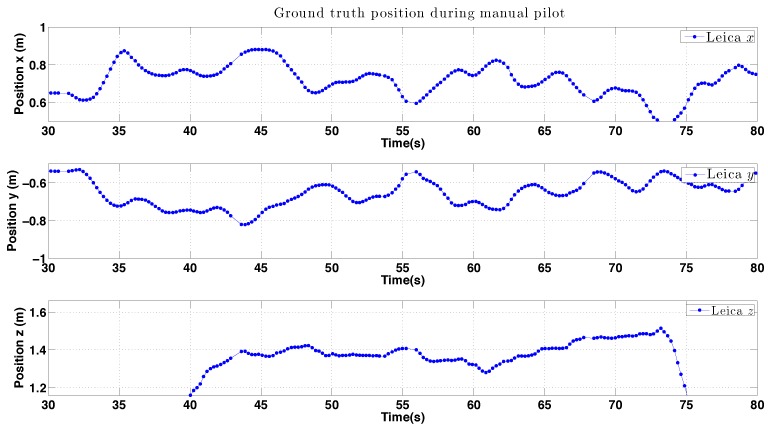
Experimental results for Pilot 2 during manual hovering: position *versus* time.

Figure 35 is a summary of the results. It compares the performance of the two pilots with that of the shared-autonomy system (PBVS and IBVS control) operated by the weaker of the two pilots. The different colors in the columns denote different experiments. We use a number of performance metrics:

Ground truth is derived from ground truth data from the laser tracker or Vicon to compute statistics of the error with respect to goal position as we have done in earlier parts of this paper. For example, Pilot 1 hovered for around 100 s and we computed $\sigma_{x}=0.079$, $\sigma_{y}=0.069$, and $\sigma_{z}=0.093$ for the period 25–80 s of the first trial shown in Figure 36. For the case of circumnavigation we compute error with respect to the path’s circle and do not penalize uneven motion around the circle.

[*A*] is the percentage pole detection rate in the onboard camera images. If the task is performed correctly the pole will be visible in 100% of the images.
[*B*] is the standard deviation of the horizontal pole centre position (pixels) in the onboard camera images. This is a more graduated performance measure than A, and says something about the quality of the translational and heading angle control of the vehicle. If the task is performed well this should be 0. Note this statistic is computed over the frames in which the pole is visible.
[*C*] is the standard deviation of the pole width (pixels) in the onboard camera images. It says something about the quality of the control of the vehicle position in the standoff direction, and if the task is performed well this should be 0. Note this statistic is computed over the frames in which the pole is visible.

**Figure 37:**
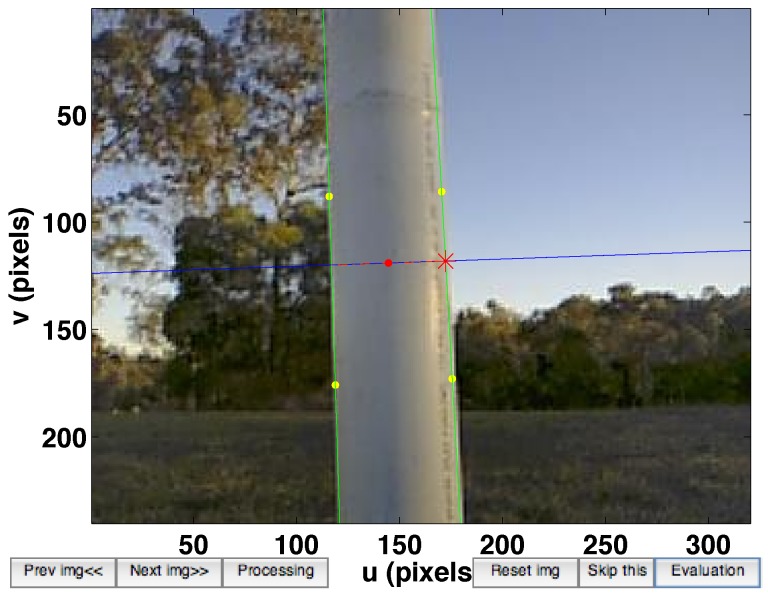
User interface of tool for generating performance metrics. The four yellow points are manually picked to define the pole edges (two green lines). The red point and star denote the calculated pole centre and width in the image coordinates respectively.

Measures *A*, *B* and *C* are computed using a semi-automated line picking software tool (shown in Figure 37) from the recorded image sequences (subsampled to 10 Hz). As shown in Figure 35, autonomous flight outperformed all manual flights. Moreover, pole detection rates (*A*) were 100% for shared autonomy circumnavigation however it decreased to 70%–80% for manually piloted flights. This is due to the fact that the pilot had to control all 4 DOFs and the heading angle had to be constantly changed during circumnavigation in order to keep the camera pointed at the pole. The cognitive load on the pilot was very high for the circumnavigation task and the task was considered to be quite stressful.

**Figure 38:**
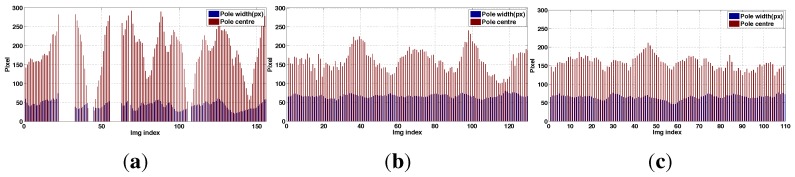
Experimental results for circumnavigation: pole width (blue) and offset of the pole centre from the image edge (red) *versus* time. Comparison of Pilot 2 manual flight (**a**), and Pilot 1 shared autonomy flights using IBVS and PBVS (**b**,**c** respectively). Since the image width is 320 pixels, a value of 160 for “Pole centre” indicates the pole was centred in the image. Zero values are for frames where the pole didn’t appear in the image. (**a**) Pilot 2; (**b**) IBVS; (**c**) PBVS.

Another interesting result was the difference in *B* and *C* for the manual hovering and circumnavigation experiments. These increased significantly from hovering to circumnavigation for manually piloted flights. For example, *B* was 13.33 pixels for Pilot 2’s first hovering trial and this increased to 76.5 pixels for circumnavigation (See Figure 35). For Pilot 1’s shared autonomy flights the results remained fairly consistent however (*B* = 12–17 pixels for PBVS, and *B* = 30 pixels for IBVS). Figure 38 also illustrates this point as we see the pole width and position in the image remain far more consistent for the shared autonomy flights (Figure 38b,c) compared to the manually piloted flight (Figure 38a). Figure 35 shows the best hovering results were achieved by IBVS (night-time) and PBVS (day-time) and best circumnavigation results were achieved by PBVS (day-time).

**Figure 39:**
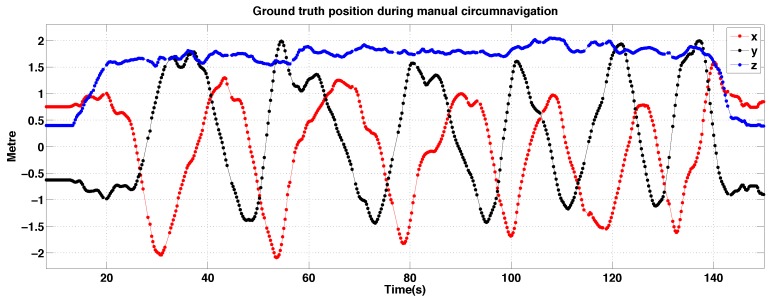
Experimental results for Pilot 2 during manual circumnavigation: position *versus* time.

**Figure 40:**
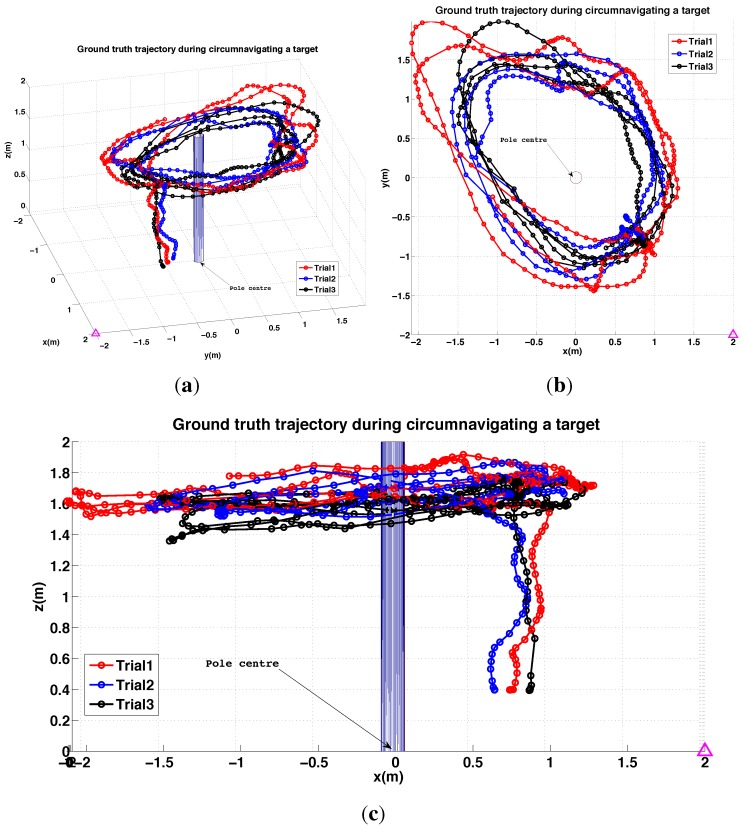
Experimental results for Pilot 2 during manual circumnavigation, 3D views. The magenta triangle indicates where the pilot was standing. (**a**) Perspective view; (**b**) Top view; (**c**) Side view.

Figure 39 and Figure 40 shows 3D position *versus* time for three circumnavigation trials by Pilot 2. The magenta triangle denotes the standing position of Pilot 2 during the circumnavigation experiments. The trajectory is elliptical in shape with its major axis passing through the standing position. We believe there are two reasons for this: firstly, it is most challenging to estimate distance along the optical axis when the only visible cue is slight change in apparent size, and secondly, the pole occluded the vehicle from the pilots’s line-of-sight at this position.

In summary the shared autonomy system allowed a less skilled pilot to achieve better task performance than our best human pilot, and at a much lower level of cognitive load and stress.

### 6.5. Limitations and Failure Cases

The proposed system has limitations which we plan to address. For example, the line tracker failed on occasion when the user commanded a large yaw rate causing the pole to leave the camera field of view (FOV). This can be addressed by either using a wider FOV lens (currently $75^{\circ }$) or by limiting the yaw rate.

**Figure 41:**
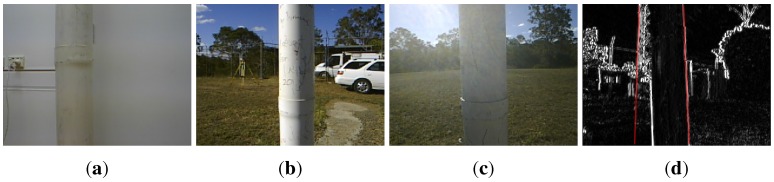
Challenging images for indoor (**a**) and outdoor (**b**–**d**) scenes.

Another limitation is the susceptibility of the line tracker to the real-world lighting effects when operating outdoors. If the sun appears in the image it leads to severe flaring and failure of the line tracker. Laser scanners are however also adversely affected when looking at the sun. Shadowing and uneven illumination in both indoor and outdoor environments, (see for example Figure 41), can create an intensity gradient on the surface of the pole and this may be falsely detected and tracked as the pole edge. To avoid these challenges our experiments were conducted in the absence of direct sunlight (early morning, late afternoon or cloudy), and we will improve the robustness to these effects in the future.

We also experienced failures when strong lines were present in the background as shown in Figure 41d. A telecommunication tower produced strong lines and the line tracker failed to track the pole.

The sonar only works reliably up to 2 m and is very noisy on grass or gravel outdoors. We therefore plan to develop a height estimator which combines other sensing modalities such as a barometer, scale from a downward looking camera or vertical visual odometry from the front camera on the pole being inspected.

**Figure 42:**
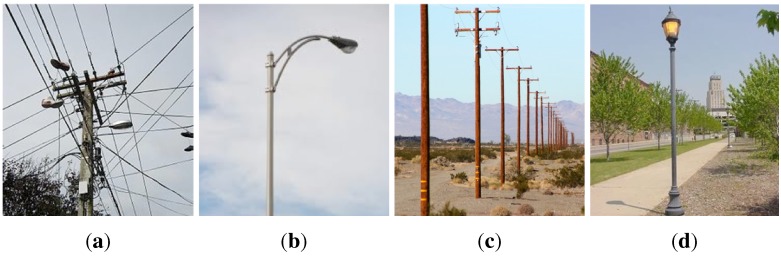
Sophisticated (**a**), curved (**b**), perceptual aliased real world power line (**c**) and street light pole (**d**) variants.

Figure 42 shows real-world vertical structures such as power line and street light poles. They are not white in colour or have the homogeneous uniform shape that we assume in this paper. Perceptual aliasing caused from identical poles placed in a row represents a common real world problem. The proposed method does not present a straightforward way of resolving these issues however the results obtained from the simplified setup we use do demonstrate its potential.

## 7. Conclusions

We have presented a VTOL platform-based pole-relative navigation system using PBVS, IBVS and shared autonomy. The target application is aerial inspection of vertical structures such as poles. The pole-relative navigation increases the autonomy of the system and facilitates the use of shared autonomy where the operator is relieved of the cognitive load of controlling all degrees of freedom. By self-regulating its stand-off distance from the pole, height, and keeping the camera facing the pole the system only requires height set points and yaw rate commands (which induce an orbit around the pole).

A common element of all the systems is an efficient and high-performance line tracker which provides estimates of the Plücker coordinate parameters of the observed lines. A key to high performance tracking on such an agile platform is feature prediction which we achieve using an image feature Jacobian and IMU measurements. For PBVS control these tracked features and IMU measurements are fed into a pose estimator of PBVS (Extended Kalman Filter) and we designed a controller based on the estimated states. IBVS control is performed directly using information from only two vertical lines (the pole edges) which leads to some unobservable and also ambiguous vehicle motions. We presented a line-feature based IBVS system which uses IMU data to eliminate the effect of body rotation and directly commands velocity in the horizontal plane.

The IBVS and PBVS systems demonstrated good pole-relative hovering and circumnavigation performance, maintaining position to within 20 cm of the goal position even in the presence off light wind.

The controllers formed part of a shared autonomy system in which the operator is no longer flying the vehicle but providing setpoints in a low DOF object-relative coordinate frame. Experimentally we showed that this allows a less skilled pilot to achieve better task performance than our best human pilot, and at a much lower level of cognitive load and stress. This sets the scene for operation of small VTOL platforms to perform cost-effective single person inspection jobs, rather than the three person crews that are currently the norm.

Finally, even though both systems demonstrate good performance, we prefer IBVS over PBVS for two reasons. Firstly, PBVS requires a pose estimator which takes image features as input and computes the metric pose of a camera. Development of a robust vision-based pose estimator that can be used in varying environments is difficult. Secondly, IBVS is relatively easy to implement since it omits the pose estimation step, and utilize the image features directly. Although IBVS can degrade observability and be poorly conditioned due to a linearization of a highly non-linear model, it works on our pole tracking application.
